# IL1β Induces Mesenchymal Stem Cells Migration and Leucocyte Chemotaxis Through NF-κB

**DOI:** 10.1007/s12015-012-9364-9

**Published:** 2012-03-31

**Authors:** Rubén Carrero, Inmaculada Cerrada, Elisa Lledó, Joaquín Dopazo, Francisco García-García, Mari-Paz Rubio, César Trigueros, Akaitz Dorronsoro, Amparo Ruiz-Sauri, José Anastasio Montero, Pilar Sepúlveda

**Affiliations:** 1 Fundación para la Investigación Hospital Universitario La Fe, Avda Campanar 21, Valencia, 46009 Spain; 2 Universidad Cardenal Herrera-CEU, Valencia, Spain; 3 Functional Genomics Node, National Institute for Bioinfortmatics, Valencia, Spain; 4 Centro de Investigación Príncipe Felipe, Valencia, Spain; 5 Fundación Inbiomed, San Sebastián, Spain; 6 Universidad de Valencia, Valencia, Spain

**Keywords:** Mesenchymal stem cells, Interleukin 1β, Chemotaxis, Migration and adhesion

## Abstract

Mesenchymal stem cells are often transplanted into inflammatory environments where they are able to survive and modulate host immune responses through a poorly understood mechanism. In this paper we analyzed the responses of MSC to IL-1β: a representative inflammatory mediator. Microarray analysis of MSC treated with IL-1β revealed that this cytokine activateds a set of genes related to biological processes such as cell survival, cell migration, cell adhesion, chemokine production, induction of angiogenesis and modulation of the immune response. Further more detailed analysis by real-time PCR and functional assays revealed that IL-1β mainly increaseds the production of chemokines such as CCL5, CCL20, CXCL1, CXCL3, CXCL5, CXCL6, CXCL10, CXCL11 and CX_3_CL1, interleukins IL-6, IL-8, IL23A, IL32, Toll-like receptors TLR2, TLR4, CLDN1, metalloproteins MMP1 and MMP3, growth factors CSF2 and TNF-α, together with adhesion molecules ICAM1 and ICAM4. Functional analysis of MSC proliferation, migration and adhesion to extracellular matrix components revealed that IL-1β did not affect proliferation but also served to induce the secretion of trophic factors and adhesion to ECM components such as collagen and laminin. IL-1β treatment enhanced the ability of MSC to recruit monocytes and granulocytes *in vitro*. Blockade of NF-κβ transcription factor activation with IκB kinase beta (IKKβ) shRNA impaired MSC migration, adhesion and leucocyte recruitment, induced by IL-1β demonstrating that NF-κB pathway is an important downstream regulator of these responses. These findings are relevant to understanding the biological responses of MSC to inflammatory environments.

**Electronic supplementary material:**

The online version of this article (doi:10.1007/s12015-012-9364-9) contains supplementary material, which is available to authorized users.

## Introduction

Mesenchymal stem cells have become a therapeutic option for several pathologies like myocardial infarction, osteogenesis imperfecta, graft versus host disease and wound healing [1–4]. As a part of the cell therapy, MSC are often transplanted in ischemic, apoptotic and/or inflammatory environments where cells survive and promote tissue regeneration by mechanisms that remain poorly understood. These cells are immunoprivileged, and in most of pathologies the induced potential benefits are related to paracrine activity mediated by their ability to survive in ischemic and inflammatory environments [5–7]. Despite their therapeutic potential initial, clinical results have been disappointing due to reported low benefits. It is believed that in adequate doses, low engraftment and poor survival are responsible for these results. We and others reported that intramyocardial MSC transplantation recruits a number of inflammatory cells that contribute to the healing of the infarct [8, 9]. Transplanted cells are consistently exposed to tissue signals, immune cells and mediators that could influence their behaviour. Since successful application of stem cell approaches will depend on the microenvironment of the recipient tissue, we have sought to investigate the response of MSC to an inflammatory environment. Previous reports showed that proinflammatory cytokines were able to increased migration of human MSC to many chemotactic factors [10], to induce MSC to produce chemokines [11] and to stimulate MSC to differentiate into neural phenotype [12]. Following this rationale we cultured MSC in the presence of inflammatory mediators and analyzed biological responses implicated in proliferation, survival, adhesion and migration that could aid to predict their response in these environments. We focused our studies in IL-1β as a prototypical inflammatory mediator and the results showed that this cytokines promotes specific biological processes in MSC in part due to activation of the transcription factor NF-κB (Nuclear Factor KAPPA-light-chain-enhancer of activated B cells).

## Materials and Methods

### Cells and Culture Conditions

Human bone marrow MSC (*n* = 4; Inbiomed, San Sebastian, Guipuzcoa, Spain) were cultured following manufacturers´ instructions. After centrifugation cells were seeded in tissue culture flasks in low glucose Dulbecco’s modified Eagle medium (Sigma-Aldrich, St. Louis, MO; htpp://www.sigmaaldrich.com), supplemented with 10% fetal calf serum (Thermo Scientific Hyclone, Rockford, IL; htpp://www.piercenet.com), 4 mM L-Glutamine (Gibco, Grand Island, NY; htpp://www.invitrogen.com) and 1% antibiotics [50U/mL penicillin, 50 μg/mL streptomycin (Gibco)]. Cells were maintained in a humidified atmosphere of 95% air and 5% CO2 at 37°C. The medium was replaced every 3 days. IL-1β (25 ng/mL), (Millipore, Temecula, CA; htpp://www.milipore.com), IL-6 (20 ng/mL), (R&D Systems, McKinley Place, MN; htpp://www.RnDSystems.com), IL-8 (20 ng/mL), (Sigma-Aldrich) and TNF-α (50 ng/mL), (Sigma-Aldrich) were added and maintained during different periods of time depending on the experiments.

### Electrophoresis and Western blotting

Cells were submitted to serum deprivation for 18 h before incubation with IL-1β (25 ng/mL) for 30 min. Cells were then washed with PBS and the monolayer lysed in M-PER® Mammalian Protein Extraction Reagent (Thermo Fisher Scientific, Rockford, IL; htpp://www.piercenet.com) containing protease inhibitors (Roche, Mannheim, Germany; htpp://www.roche.com)] and phosphatase inhibitors [Sodium Orthovanadate 1 mM (Sigma-Aldrich), Sodium Fluoride 1 mM (Sigma-Aldrich)]. Protein concentration was quantified using the Pierce® BCA Protein Assay Kit (Thermo Fisher Scientific). For western blotting 30 μg of protein was loaded and separated by 10% SDS-PAGE, before transfer to a PVDF membrane (Thermo Scientific) and blockinged with 5% BLOT-Quick Blocker (Millipore). Primary antibodies were incubated at 4°C overnight. After, the blots were incubated with IgG HRP Conjugated for 1 h at room temperature. Detection was performed with ECL system [ECL Plus^TM^ Western Blotting Detection Reagents Amersham (GE healthcare, Bukinghamshire, UK; htpp://www.gehealthcare.com)]. GAPDH was used to determine equal protein loading. Antibodies used were anti-phospho-Akt 1/2/3 (Ser473), anti-phospho-NFκβ p65 (Ser536) (Cell Signaling Tecnology Inc., Danvers, MA; htpp://www.cellsignaling.com), anti-GAPHD, anti-ERK 1/2 (MAPK), anti-phospho-ERK 1/2 (MAPK), (Chemicon, Temecula, CA; htpp://www.chemicon.com), anti-Akt 1/2/3, anti-NFκβ(p65), anti-rabbit IgG HRP Conjugated (Santa Cruz Biotecnology Inc., Santa cruz, CA; htpp://www.scbt.com) and anti-mouse IgG HRP Conjugated (Promega, Madison, WI; htpp://www.promega.com). All antibodies used were assayed at 1:1,000 dilution except anti-Akt 1/2/3 and anti-NFκB (p65) that were used at 1:500.

### Cell Cycle Assay

To analyze the effect of IL-1β in cell cycle, 10^6^ cells were harvested, fixed with 70% EtOH and kept at −20°C until use. Fixed cells were centrifugated and resuspended in 50 μg/mL propidium bromide (Sigma-Aldrich) and analyzed by flow cytometry (488 nm excitation, 625 nm emission).

### MTT Proliferation Assay

Cell proliferation was determined using a 3-(4, 5-dimethylthiazol-2-thiazolyl)-2,5-diphenyl-2H-tetrazolium bromide (MTT) assay. MSC were plated at a density of 10^4^ cells/cm^2^ in 96-well microplates. 24 h post seeding, cells were treated with recombinant IL-1β (25 ng/mL) or IL-6 (20 ng/mL) for 24 h. Proliferation was assayed by Thiazolyl Blue Tetrazolium Bromide (Sigma-Aldrich), acording to manufacturer´s instructions. The absorbance of the samples was measured at 570 nm using a microplate reader (Victor3 1420 Multilabel Counter; PerkinElmer Inc., Waltham, MA; htpp://www.perkinelmer.com). Experiments were performed in triplicate and results were expressed relative to the untreated control.

### Construction of IKKβ shRNA and Lentiviral Transduction

IKKβ shRNA secuences have been published previously [13] and were purchased from Sigma-Genosys (Sigma-Aldrich): - IKKβi: GGAAGTACCTGAACCAGTTTG. Oligos were annealed and cloned into pSUPER plasmid carrying H1 promoter using BglII-HindIII sites. The H1-shRNA expression cassette was then excised and cloned into pLVTHM (Addgene plasmid 12247; Addgene, Cambridge, MA, http://www.addgene.org) using EcoRI-ClaI sites. Viral particles were produced in human embryonic kidney 293 T cells (ATCC, www.atcc.org). Briefly, 293 T cells were seeded in high glucose DMEM containing 10%FBS. psPAX (Addgene plasmid), pVSV-G (Addgene plasmid 12259, www.addgene.org) and the lentiviral vector pLVTHM containing GFP reporter gene and shRNA sequences were transfected in to the packing cells using calcium phosphate precipitation method. Viral transduction was carried out using a multiplicity of infection (M.O.I.) of 10 in the presence of 8 μg/mL of polybrene (Sigma-Aldrich) in order to achieve 100% infection.

### MSC Migration Assay

To study trophism induced in MSC by IL-1β, cells were seeded in basal medium (DMEM with 0.5% FBS) at 10,000 cells/cm^2^ in the top chamber of an 8 μm-pore migration transwell (BD Falcon, Bedford, MA, htpp://www.bd.com). After overnight culture, 25 ng/mL of IL-1β was added to the bottom chamber and cells were fixed with 2% paraformaldehide (Panreac Química, Castellar del Vallés, Spain), SDF-1α (20 ng/mL) and 10% FBS were used as positive controls. Briefly, after 4 h non migrated cells were removed from the upper side of the membrane using a cotton bud to remove non migrating cells, the membrane was cut and placed in a glass slide with the bottom side upwards and before staining with 4´,6 diamidino-2 phenyilindole (DAPI) (Sigma-Aldrich). All assays were performed in duplicated wells and repeated three times. Migrated cells were counted as fold increase relative to passive MSC cell migration in untreated wells.

### Leucocyte Migration Assay

To determine the nature of human leucocytes that could be recruited in response to paracrine factors secreted by MSC, we established co-culture of MSC and pheripheral blood leucocytes (PBLs) using a transwell culture system (BD Falcon). MSC (10,000 cells/cm^2^) seeded in the lower chamber of the transwells were stimulated or not with IL-1β for 2 h. After extensive washes with PBS, wells were filled with fresh medium and human PBLs from buffy coats (100,000 cells) were seeded in the upper chamber. Migrated cells through 8 μm-pore size membranes were counted after 5 h of co-culture. Cells were fixed as described above and leucocyte populations were quantified in hematoxilin stained preparation by morphologic counting. All studies were performed in a blinded fashion.

### Adhesion Assay

Cells were seeded in basal medium onto cover slides previously treated with 10 μg/cm² of fibronectin (Sigma-Aldrich), 2 μg/cm² of laminin (Sigma-Aldrich) and 10 μg/cm² of collagen (Sigma-Aldrich). After 1 h, cells were fixed with 2% paraformaldehyde, washed with PBS, labelled with 4´,6-diamidino-2-phenylindole (DAPI) and counted.

### Reverse Transcription and Real-Time Quantitative PCR

MSC incubated in different conditions were washed with PBS. RNA was extracted using TRIzol Reagent (Invitrogen, Carlsbad, CA; htpp://www.invitrogen.com) and purified with RNeasy Plus Mini Kit (Qiagen, Dusseldorf, Germany; htpp://www.qiagen.com). RNA samples were quantified by spectrometry (NanoDrop ND-1000, NanoDrop Technologies, Wilmington, DE; htpp://www.nanodrop.com) and integrity was assessed by agarose gel electrophoresis and the absorbance ratio 260/280 nm. cDNA was obtained by retrotranscriptase reverse transcription using M-MLV Reverse Transcriptase (Invitrogen) from total RNA (1 μg). Primers were designed using the Primer-Blast online tool (Table 1). The Ct of each PCR in a reference human MSC cDNA sample (dilution 1/10) is expressed as mean ± standard deviation (SD) of three independent PCR experiments. Real-time PCR was performed using convenient primers and SYBR Green I [1X LightCycler 480 SYBR Green I Master (Roche Molecular Biochemical, Mannheim, Germany; htpp://www.roche.com)]. Plates were run in a real-time thermal cycler (LightCycler 480 Instrument; Roche Diagnostics, Mannhein, Germany, htpp://www.roche.com) following manufacturer´s instructions. Real-time monitoring of the PCR reaction was performed with the LightCycler 480 Software 1.5, as well as the quantification of the products in the exponential phase of the amplification. Relative expression levels were calculated with the Relative Quantification Analysis software. Results were considered to be significant with a 2-fold induction. For all real-time experiments, gene expression levels were normalized to two human housekeeping genes ACTB and GAPDH and average from triplicate samples.

**Table 1:** List of oligonucleotides used for Real time-PCR. Ct and efficiency values are indicated

Gene	Forward primer	Reverse primer	Size (bp)	Ct	SD Ct	Efficency^a^
ACTB	AGAGCCTCGCCTTTGCCGATCC	CATGCCGGAGCCGTTGTCGAC	101	16.10	0.025	1.971
CCL2	TCTCAGTGCAGAGGCTCGCGA	CCACTTCTGCTTGGGGTCAGCAC	119	17.01	0.006	1.977
CCL3	TTCAGAAGGACACGGGCAGCAGACA	GGAATCTGCCGGGAGGTGTAGCT	216	29.64	0.050	1.862
CCL5	TACATTGCCCGCCCACTGCC	GGGTTGGCACACACTTGGCG	119	23.82	0.052	2.061
CCL7	CAGCTGCTTTCAGCCCCCAGG	GCTTCCCGGGGACAGTGGCTA	148	23.93	0.032	1.923
CCL8	TGGCAGCCACTTTCAGCCCT	GCACAGACCTCCTTGCCCCG	190	27.98	0.167	2.040
CCL20	TCTGCGGCGAATCAGAAGCAGC	TTCATTGGCCAGCTGCCGTGT	110	21.19	0.068	2.16
CLDN1	CCGGGTTGCCCACCTGCAAA	CGTACATGGCCTGGGCGGTC	258	24.49	0.148	1.862
CLDN14	GTCGCTGTGGGCAGGTGGTC	AGCCTCCCCTTCCCAGCCTG	192	30.48	0.093	2.084
CSF2	TGCAGCATCTCTGCACCCGC	AGGCAGGTCGGCTCCTGGAG	176	24.47	0.188	1.708
CSF3	AGACCCATGGCTGGACCTGCC	GTGGCACACTCACTCACCAGCTTC	218	31.56	0.043	2.026
CX3CL1	CTGGCTGGACAGCACCACGG	GCTCCTTCGGGTCGGCACAG	175	25.96	0.087	1.880
CXCL1	AGCCTGCAACATGCCAGCCA	TGTGCACATACATTCCCCTGCCT	94	21.72	0.036	1.848
CXCL10	TGCAAGCCAATTTTGTCCACGTGT	GCAGCCTCTGTGTGGTCCATCC	200	24.10	0.051	1.956
CXCL11	TGTCTTTGCATAGGCCCTGGGGT	AGCCTTGCTTGCTTCGATTTGGGA	164	29.72	0.178	1.821
CXCL3	AATGTAAGGTCCCCCGGACCCC	ACCACCCTGCAGGAAGTGTCAA	199	20.92	0.036	2.023
CXCL5	AATCTCCGCTCCTCCACCCAGT	GCTCTCTCAACACAGCAGCGGC	201	28.49	0.050	1.614
CXCL6	GCACGAGGAAACCAAAGTGCTCTG	GTGCAACGCAGCTCTGTCAGCA	234	26.37	0.071	1.890
ELF3	ATTTAGAGCCGGGTAGGGGAGCG	GTTGCAGCCATGAGGCTACCGGAGT	132	25.68	0.088	1.912
GAPDH	CCCCTCTGCTGATGCCCCCA	TGACCTTGGCCAGGGGTGCT	122	16.79	0.015	1.998
IBSP	GGAGTACGAATACACGGGCGCC	GGTAGCCGGATGCAAAGCCAGA	222	27.12	0.049	2.018
ICAM1	CTGGTCCTGCTCGGGGCTCT	GGGCTGGTCACAGGAGGTGC	126	22.42	0.094	1.638
ICAM4	AATACACTTTGCGCTGCCACGTG	GGCTCCAAGCGAGCATCAGTGT	264	28.16	0.116	2.057
IL11	TGACCCGCTCTCTCCTGGCG	GCACGTGCCGCAGGTAGGAC	192	24.23	0.163	1.803
IL12A	CCCAAAACCTGCTGAGGGCCG	TGGAGGCCAGGCAACTCCCAT	219	29.08	0.047	1.985
IL15	GCTGCTGGAAACCCCTTGCCAT	TAGGTGCTTTGGGCCAACTGGG	214	28.78	0.035	1.899
IL1B	AGGCACAAGGCACAACAGGCT	AACAACTGACGCGGCCTGCC	277	21.12	0.110	1.965
IL23A	TGGCTGTGACCCCCAAGGACTC	TGCCATGGCTGGCTGGGACT	246	27.01	0.053	1.893
IL32	TTTGTGCCAGGAAGACTGCGTGC	GGCTCGACATCACCTGTCCACG	215	24.41	0.078	2.004
IL34	GAACACCACCATGCCCCGGG	CAGCCTGGTGACGTTGGCGA	250	24.59	0.078	1.853
IL6	CATTCTGCCCTCGAGCCCACC	GGCAGCAGGCAACACCAGGA	139	17.08	0.088	1.955
IL7	GCTGCTCGCAAGTTGAGGCAATTT	TTGTTGGTTGGGCTTCACCCAGG	157	26.58	0.213	1.974
IL8	CGTGGCTCTCTTGGCAGCCTTC	TTCCTTGGGGTCCAGACAGAGCTC	229	15.88	0.707	2.005
ITGA9	GCAGTGACCGCTGGCCACTT	GCGCACAAGGAGGAGCCGAA	182	27.63	0.098	1.969
ITGB3	TGCCGCCCTGCTCATCTGGA	TCCTGCAATCGTGGCACAGGC	239	22.43	0.008	2.192
ITGB8	CTAGCGACACTCGGCCCGC	CTGGACCCAGCGCAAGGCAC	292	27.76	0.023	1.850
MMP1	GTGTCTCACAGCTTCCCAGCGAC	GCACTCCACATCTGGGCTGCTTC	238	25.01	0.084	2.006
NOD2	AGGCCTACCCGCAGATGCCA	GTGGGAGAGAGGCTGGCCCA	298	28.04	0.172	2.062
SDC4	CGGAGTCGCCGAGTCGATCC	GGCTCCCAGACCCTGCCCTC	248	20.51	0.030	1.925
SELECTIN	TTGTTCCTGCCAGCAGCTGCC	AGGGCCAGAGACCCGAGGAG	164	17.64	0.085	2.030
SERPINE1	AGGACGAACCGCCAATCGCAAG	ACCCTCACCCCGAAGTCTGAGG	167	25.86	0.034	2.120
TCAM1P	CGAGCTTGGCTGTGGCCTCC	TCTCCGCCATCCCAGCCTCC	225	27.05	0.078	1.856
TLR2	AGGCAGCGAGAAAGCGCAGC	CCCCCAAGACCCACACCATCCA	253	31.23	0.352	2.162
TLR4	CCCTGCGTGGAGGTGGTTCCTA	CTCCCAGGGCTAAACTCTGGATGGG	280	26.45	0.024	2.007
TNF	CCCTCTGGCCCAGGCAGTCA	ATGGGTGGAGGGGCAGCCTT	235	21.58	0.019	2.100
VCAM1	AGGTGACGAATGAGGGGACCACA	CCAGCCTCCAGAGGGCCACT	181	17.31	0.010	2.039

^a^PCR efficiency of each PCR was estimated from a serially diluted standard curves obtained from a reference MSC cDNA sample. The cycle number of the crossing point CP vs. Log (dilution factor) were plotted to calculate the slope. The efficiency of each PCR was then calculated with the equation ${\hbox{E}} = {1}{0^{[ - {1}/{\rm{slope}}]}}$

### Microarray Assays

Human bone marrow MSC were treated or not with IL-1β (25 ng/mL) for 24 h at 37°C in a humidified incubator with 5% CO2. Cells were collected and total RNA was extracted using the High Pure RNA Isolation kit (Roche) and quantified by spectrometry (NanoDrop ND-1000, NanoDrop Technologies). 800 ng of total RNA were used to produce Cyanine 3-CTP-labeled cRNA using the Low RNA Input Linear Amplification Kit PLUS (Agilent, Santa Clara, CA¸ http://www.chem.agilent.com). Based on the protocol for One-Color Microarray-Based Gene Expression Analysis Version 5.5 (Agilent p/n 5188–5977), 3 μg of labeled cRNA was hybridized with Whole Human Genome Oligo Microarray Kit (Agilent p/n G4112F) containing 41,000+ unique human genes and transcripts. Arrays were scanned in an Agilent Microarray Scanner (Agilent G2565BA) according to the manufacturer’s protocol and data extracted using Agilent Feature Extraction Software 9.5.1. Hybridization was performed by the microarray core facility from Centre Principe Felipe.

### Microarray Data Analysis

Signal was standardized across arrays using quantile normalization [14]. Differential gene expression was carried out using the fold change. Gene set analysis was carried out for the Gene Ontology terms using FatiScan [15] from Babelomics web tool [16]. GO annotation for the genes in the microarray where taken from Ensembl 55 release (http://www.ensembl.org, Ensembl org, Hinxton, UK), allowing the visualization of functional categories within their biological context. Results were considered to be significant with a 2-fold induction.

The microarrays data of this study have been deposited in the Gene Expression Omnibus database under accession number GSE33755.

### Statistical Analysis

Data are expressed as mean ± SD. Comparisons between experimental groups were done with unpaired and paired two-samples t-test using the SPSS software (SPSS, Chicago, IL http://www.spss.com). Differences were considered statistically significant at *P* < 0.05.

## Results

### Global Transcriptome Profiling of MSC Cultured with IL-1β

To test the effect of IL-1β on MSC, cells were cultured with or without 25 ng/mL of IL-1β for 24 h. Gene expression changes induced by the pro-inflammatory cytokine were evaluated by microarray analysis. Further bioinformatics analysis using Babelomics software (http://www.babelomics4.org) was performed to classify genes by function using the Gene Ontology (GO) scheme, revealing the main families of genes affected by the treatment. Growth in IL-1β resulted in activation of genes associated to very specific GO categories. In particular, we identified alterations in the expression of genes implicated in the following biological processes: i) response to wounding, ii) immune and inflammatory response, iii) defense response, iv) chemotaxis, v) locomotory behaviour, vi) regulation of cell proliferation, vii) leukocyte chemotaxix, viii) I-kappaB kinase/NF-kappaB cascade, ix) negative regulation of apoptosis, x) blood coagulation, and xi) cell adhesion (Table 2). Fold changes of up-regulated genes (negative values) from enriched biological processes in MSC treated with IL-1β (MSC-IL1β) are indicated (Supplemental Table 1).

**Table 2:** Enriched biological processes for up-regulated genes in MSC-IL1β versus MSC

GO biological process	Process gene set	*P* value
GO:0009611	Response to wounding	2.00E-21
GO:0006955	Inmune response	1.74E-19
GO:0006954	Inflammatory response	2.43E-18
GO:0006952	Defense response	2.43E-17
GO:0006935	Chemotaxis	5.44E-10
GO:0007626	Locomotory behavior	6.15E-9
GO:0042127	Negative regulation of cell proliferation	1.27E-5
GO:0030595	Leukocyte chemotaxis	3.04E-3
GO:0007249	I-kappaB kinase/NF-kappaB cascade	3.27E-3
GO:0043066	Negative regulation of apoptosis	3.64E-3
GO:0007596	Blood coagulation	1.36E-2
GO:0007155	Cell adhesion	3.26E-2

### IL-1β Increases Expression of Multiple Chemokines and Growth Factors in MSC

**Fig. 1:**
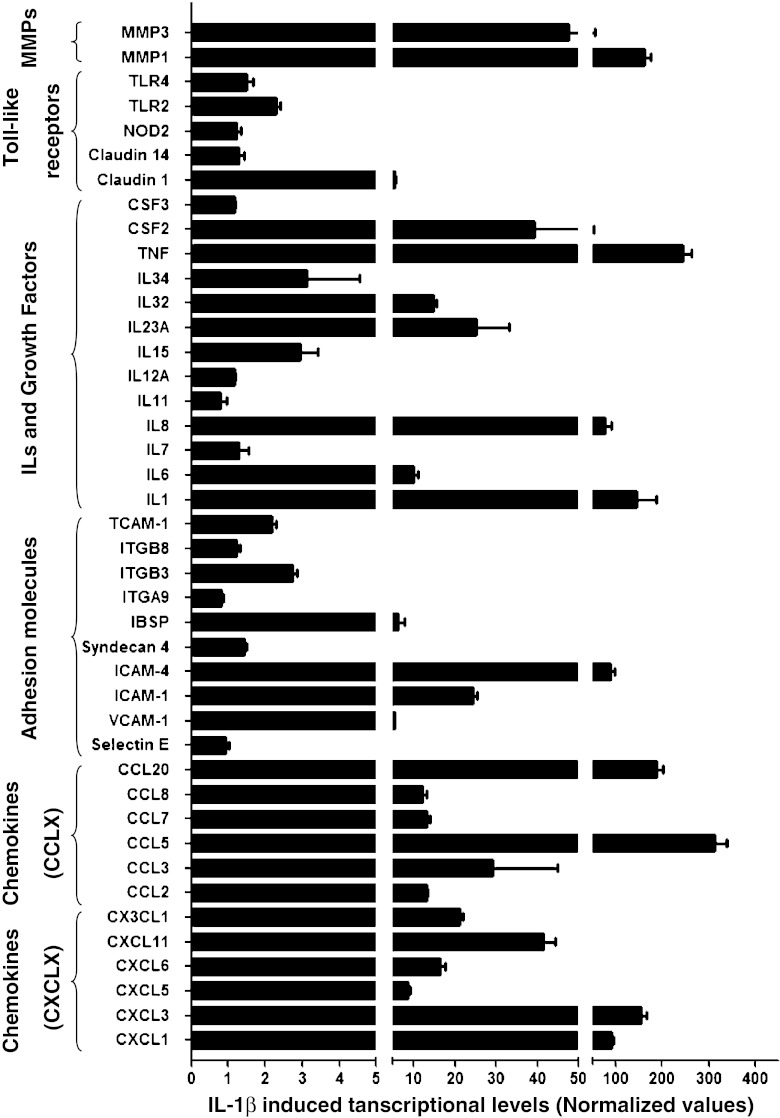
Quantification of gene transcription levels by qRT-PCR analysis in MSC cultures treated with IL-1β versus untreated cultures. Data are expressed as relative fold of control (normalized values) and represent the mean ± S.D. of three independent experiments

After bioinformatic analysis, highly up-regulated genes related with these biological processes were further assayed by real-time PCR (Fig. 1, Table 3). Chemokines are small molecules that direct the migration of immune cells via chemokine-chemokine receptor interactions. Based on their genetic organization and the position of two highly conserved cysteine residues at the N-terminus, chemokines can be divided into four subgroups, the CC, CXC, C, and CX_3_C families [17]. Among CC chemokines, CCL5 and CCL20 were the most up-regulated in response to IL-1β treatment (312 ± 27 and 187 ± 15 fold, respectively). CXCL1, CXCL3 and CX_3_CL1 were also highly expressed after IL-1β treatment. CXCL10, CXCL11 and ELF3 were expressed de novo upon stimulation of MSC with IL-1β (Table 3). Regarding the cell adhesion molecules, the most important families include the intercellular adhesion molecules (ICAMs), the vascular adhesion molecules (VCAMs), cadherins, and selectins. Among them, IL-1β treatment increased expression of the integrin binding sialoprotein (IBSP), ICAM1, ICAM4, integrin beta 3 platelet glycoprotein IIIa (ITGB3), TCAM1P and VCAM1 as detected by real-time PCR (Fig. 1 and Table 3). Other adhesion molecules also showed a significant fold detected by microarray analysis (Supplemental Table 1).

**Table 3:** Transcriptional levels of up-regulated genes in MSC after treatment with IL-1β. Normalized values of MSC-IL1β vs non-treated MSC were calculated and expressed as mean ± standard deviation (SD) of three independent PCR experiments

Chemokines	Genebank number	Mean Ct	SD Ct	Normalized values	Normalized Error
CXCL1	NM_001511	21.72	0.036	89.1	5.9
CXCL3	NM_002090	20.92	0.036	154	11
CXCL5	NM_002994	28.49	0.050	8.65	0.59
CXCL6	NM_002993	26.37	0.071	16.5	1.27
CXCL10	NM_001565	24.10	0.051	2028	127
CXCL11	NM_005409	29.72	0.178	41.3	3.07
CX3CL1	NM_002996	25.96	0.087	21.1	0.76
CCL2	NM_002982	17.01	0.006	13.1	0.46
CCL3	NM_002963	29.64	0.050	29.2	15.8
CCL5	NM_002985	23.82	0.052	312	27
CCL7	NM_006273	23.93	0.032	13.1	0.90
CCL8	NM_005623	27.98	0.167	12.2	1.00
CCL20	NM_004591	21.19	0.068	187	15
Adhesion molecules					
Selectin E	NM_000450	17.64	0.085	0.94	0.08
VCAM-1	NM_001078.3	17.31	0.010	5.33	0.19
ICAM-1	NM_000201	22.42	0.094	24.5	0.84
ICAM-4	NM_001544.3	28.16	0.116	87.4	9.1
Syndecan 4	NM_002999.3	20.51	0.030	1.45	0.05
IBSP	NM_004967.3	27.12	0.049	6.21	1.64
ITGA9	NM_002207.2	27.63	0.098	0.81	0.05
ITGB3	NM_000212.2	22.43	0.008	2.75	0.11
ITGB8	NM_002214.2	27.76	0.023	1.24	0.07
TCAM-1	NR_002947.1	27.05	0.078	2.17	0.12
Interleukines					
IL1	NM_000576.2	21.12	0.110	145	41
IL6	NM_000600.3	17.08	0.088	9.99	1.12
IL7	NM_000880.3	26.58	0.213	1.29	0.27
IL8	NM_000584.3	15.88	0.707	76	14.4
IL11	NM_000641.2	24.23	0.163	0.79	0.18
IL12A	NM_000882.3	29.08	0.047	1.16	0.05
IL15	NM_172175.2	28.78	0.035	2.94	0.48
IL23A	NM_016584.2	27.01	0.053	25.3	7.8
IL32	NM_001012631.1	24.41	0.078	14.8	0.81
IL34	NM_152456.2	24.59	0.078	3.12	1.44
Growth factors					
TNF	NM_000594	21.58	0.019	243	21
CSF2	NM_000758	24.47	0.188	39.3	13.2
CSF3	NM_000759	31.56	0.043	1.16	0.03
ELF3	NM_004433	25.68	0.088	656	45
Toll-like receptors					
Claudin 1	NM_021101.4	24.49	0.148	5.49	0.23
Claudin 14	NM_144492.2	30.48	0.093	1.30	0.13
NOD2	NM_022162.1	28.04	0.17	1.23	0.11
TLR2	NM_003264.3	31.23	0.352	2.29	0.12
TLR4	NM_138554.3	26.45	0.024	1.50	0.16
Metalloproteins					
MMP1	NM_002421.3	25.01	0.084	162	12
MMP3	NM_002426.4	16.56	0.650	47.5	7.02

Treatment with IL-1β influenced the secretion of interleukins and growth factors. The highest differences in fold change were found in TNF-α, followed by IL-8 and CSF2 levels (Fig. 1 and Table 3). Whereas TNF-α is a master inflammatory cytokine implicated in many biological process, IL-8 and CSF2 have more restricted biological activities. Indeed, IL-8 has been predominantly associated to chemotaxis of neutrophils [18] whereas CSF2 is implicated in monocytic differentiation [19].

Other biological processes activated in response to IL-1β were related to host defence and immune response. Microarray analysis and real time PCR experiments showed up-regulation of several Toll-like receptors (TLRs), claudins and NOD proteins. These molecules are implicated in the innate immune response to microbial infection. However, recent reports have revealed that these molecule also modulates biological processes in MSC such as differentiation, migration and immunomodulatory responses [20, 21].

### IL-1β Activates the NF-κB Pathway and do not Induce MSC Proliferation

**Fig. 2:**
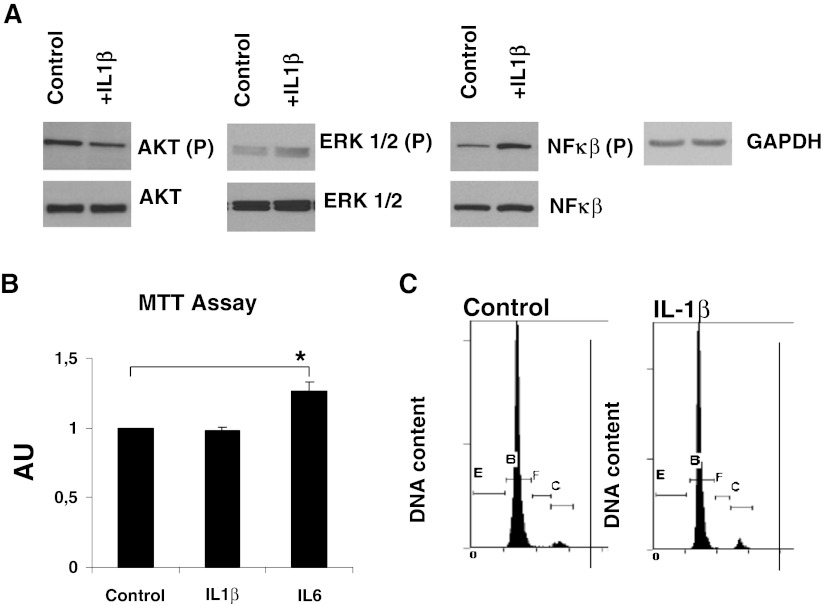
**a**, Western blot analysis of kinase phosphorylation in MSC treated with IL-1β. Total cell lysates (30 μg) were separated by SDS-PAGE. Anti-GAPDH was used as loading control. **b**, MTT assay of MSC cultures treated with IL-1β (25 ng/mL) and IL-6 (20 ng/mL) for 24 h. Values are expressed in fold increase relative to control. **c**, Cell cycle analysis by flow cytometry of MSC cultures treated with IL-1β (25 ng/mL) for 24 h. (*P < 0.05)

We next analyzed the effect of IL-1β on BM-MSC signal transduction and cell proliferation. IL-1β promoted phosphorylation of NF-κB, but not PI3K/AKT and ERK1/2 pathways (Fig. 2a), as reported for other cell types [22]. However, in correlation with the result of the microarray analysis (Table 2), IL-1β did not induce significant cell proliferation as assessed by MTT assay (Fig. 2b). These results were further confirmed by cell cycle analysis using flow cytometry (Fig. 2c). The percentage of cells in G0-G1 was 91.16 ± 2.71 in MSC versus 90.19 ± 2.72 in MSC- IL-1β. The percentage of cells in S phase was 2.28 ± 1.21 in MSC versus 1.60 ± 0.46 in MSC- IL-1β, and finally the percentage of cells in G2-M phase was 5.43 ± 1.16 versus 6.40 ± 1.69, respectively.

### IL-1β Induced Migration and Adhesion of MSC is Mainly Activated Through NF-κB Signaling

**Fig. 3:**
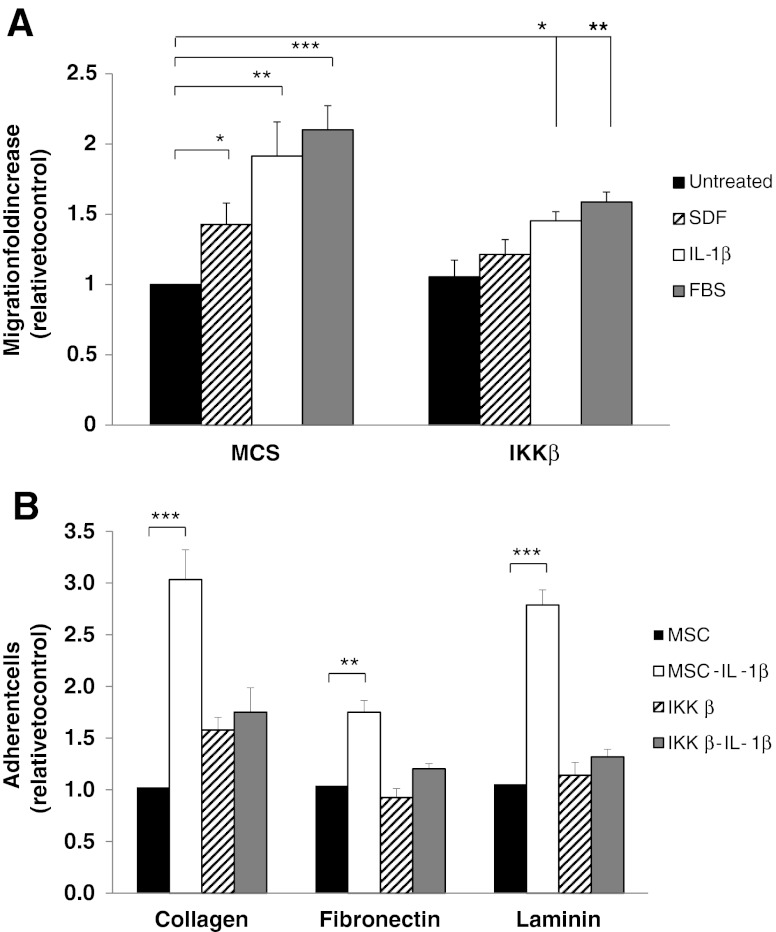
**a**, migration of MSC or MSC-IKKβ towards trophic factors SDF-1α (20 ng/mL), IL-1β (25 ng/mL) and 10% FBS. **b**, Adhesion of untreated MSC (black bars) and MSC-IKKβ (*dashed bars*) or treated with IL-1β (white and grey bars, respectively) to collagen, fibronectin and laminin. Data are represented as fold increase relative to MSC control. (*P < 0.05, **P < 0.01, ***P < 0.001 in both panels)

We and others have previously described that MSC are able to migrate *in vivo* to ischemic and pro-inflammatory environments [8, 23, 24] and it is believed that this behaviour may underlie the ability of these cells to accelerate wound healing. Migration of MSC towards cytokines, chemokines and growth factors has also been explored *in vitro* [10]. To test if IL-1β was able to increase migratory ability in MSC, we cultivated MSC in the upper chamber of a transwell and stimulated migration by adding SDF-1α, IL-1β or 10% FBS in the lower chamber (Fig. 3a). A negative control of for migration was achieved using the same proportion of fetal bovine serum (0.5% FBS) in the upper and lower chamber. SDF-1α was used since it is a well-known trophic factor for MSC implicated in homing to ischemic areas [24], and 10% FBS was used as positive control since it is a rich source in cytokines and growth factors. Surprisingly, the migratory response of MSCs to IL-1β was in fact more pronounced than it was to to SDF-1α (1.68 ± 0.21 fold increase versus 1.35 ± 0.16), indicating a strong promigratory role for IL-1β Maximum migration was achieved towards FBS gradient (1.87 ± 0.12 fold increase). Similar levels of cell migration were observed when TNF-α or IL-8 were used as trophic factors (not shown), indicating that multiple inflammatory mediators can exert trophic effects on MSC as reported [24]. We next wanted to investigate whether the signaling pathways induced by IL-1β could be directly linked to MSC migration towards trophic factors. NF-κB transcription factors play an important role in the balance between cell survival and apoptosis and are involved in the regulation of cell proliferation and differentiation of various cell types [25]. IKKβ phosphorylates IκB molecules, the inhibitors of NF-κB, leading to ubiquitination and proteasome degradation of the inhibitors, and hence release and activation of NF-κB [26]. NF-κB has previously been described as the main transcription factor activated in many pro-inflammatory responses [27]. In these context, regulation of NF-κB cascade members was observed among the biological processes most positively affected by IL-1β treatment (Table 2) and phosphorylation of NF-κB was induced on MSC after IL-1β treatment (Fig. 2). Thus, we sought to evaluate the role of NF-κB signaling in the biological responses of MSC in response to IL-1β. For this purporse, we constructed a vector containing shRNA targeting IKKβ that was lentiviraly transduced in MSC. We then evaluated the migratory response to IL-1β, SDF-1α and FBS. As shown in Fig. 3a, treatment with IKKβ shRNA reduced trophic response of MSC towards each of the 3 trophic factors assayed. An increase in the basal response of IKKβ transduced cells of 1.05 ± 0.11 fold was observed, and in response to trophic factors this was increased by 1.21 ± 0.11 towards SDF-1α, 1.45 ± 0.06 towards IL-1β, and 1.58 ± 0.07 towards 10% FBS, strongly suggesting that NF-κB signaling pathway plays a major role in MSC trophism.

**Fig. 4:**
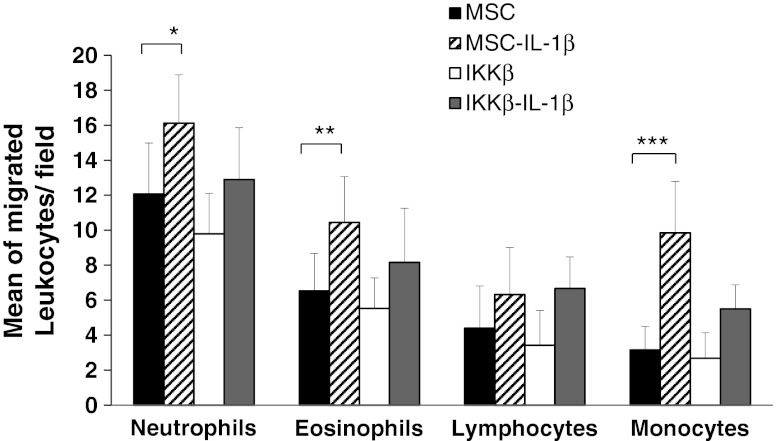
Leucocyte migration assay of human PMNs using a transwell insert culture system towards different stimuli. Upper chamber were filled with PMNs and lower chambers were seeded with MSC (*black bars*) or MSC-IKKβ (*white bars*) treated (dashed and grey bars, respectively) or not with IL-1β. Migrated neutrophils, eosinophils, lymphocytes and monocytes are presented as a mean of three experiments ± S.D. (number of migrated cells/field). *P < 0.05, **P < 0.01, ***P < 0.001

Migration and invasiveness of adherent cells is in part mediated by changes in the affinity of cells to particular ECM components (ECM). To test whether IL-1β had an effect on MSC cell adhesion, we measured the adhesion of MSC to the main components of ECM. The results showed that IL-1β treatment increased the adhesion to collagen (3.03 ± 0.29 fold), fibronectin (1.75 ± 0.11 fold) and laminin (2.79 ± 0.15 fold) (Fig. 4b). In similar way to migration experiments, adhesion induced by IL1β treatment to collagen (1.75 ± 0.15 fold), fibronectin (1.20 ± 0.05 fold) and laminin (1.32 ± 0.07 fold) was impaired in IKKβ-MSC. The fact that IKKβ expression only affected the adhesion induced by IL-1β but not the basal levels of adhesion to extracellular matrix components indicates that IKKβ blocks specifically the mechanisms induced by this cytokine, confirming the importance of NF-κB signaling pathway in the IL-1β mediated biological processes.

### Il-1β Treatment of MSC Increases Recruitment of Leucocytes In Vitro

MSC have been shown to recruit inflammatory cells such as neutrophils, eosinophils, macrophages and to suppress proliferation of cytotoxic and helper T cells through the release of soluble factors such as HGF and TGF-β [11, 28–30]. Moreover, infusion of MSC into myocardium and other tissues is accompanied by marked, paracrine mediated leucocytic infiltration [4, 8]. In order to test whether IL-1β treatment had a similar impact in MSC leucocyte recruitment, we cultured control or IL-1β treated MSCs, in a transwell system and measured the number and the type of leucocytes that migrate through a the 8 μm pores of the membrane. After 5 h of culture, the mean number of migrated leucocyes/field towards the MSC lower chambers were; 12.06 ± 2.91 neutrophils, 6.52 ± 2.14 eosinophils, 4.40 ± 2.41 lymphocytes and 3.15 ± 1.34 monocytes. However, migration towards IL-1β treated MSCs increased the number of migrated neutrophils (16.11 ± 2.75, *P* < 0.05), eosinophils (10.44 ± 2.61, *P* < 0.01), lymphocytes (6.31 ± 2.68, n.s.) and monocytes (9.85 ± 2.94, *P* < 0.001) (Fig. 4). The observed increase in chemotactic leucocyte migration induced by IL-1β treated MSC was again impaired when MSC were transduced with IKKβ (9.78 ± 2.29 neutrophils, 5.52 ± 1.74 eosinophils, 3.42 ± 1.98 lymphocytes and 2.68 ± 1.45 monocytes). Thus, IL-1β induced recruitment of neutrophils, eosinophils and monocytes and NFκB plays a mayor role in trophism exerted by MSC on these cell populations.

## Discussion

MSC have been used to treat a wide variety of diseases. Whilst the contribution of differentiation/transdifferentiation to tissue repair, are often minimal, other positive angiogenic and immunomodulatory effects are exerted by MSC in ischemic, apoptotic and pro-inflammatory environments [6].

IL-1β is produced in different tissues, not only as a response to pathogens, but also as a danger signals in pathologies such as acute myocardial infarction [31], type 2 diabetes [32], neural disorders [33]. In this study we wanted to investigate the response of MSC to proinflammatory stimuli in terms of survival, proliferation and induced paracrine factors. Thus, we treated MSC with IL-1β and used microarray to infer the biological response, firstly by gene function and later by direct gene set with known functional outcomes. A range of biological responses were activated in response to IL-1β, but perhaps, the most prominent was the potent stimulation of secretion of chemokines and growth factors that in turn were able to increase migration and adhesion of MSC and to regulate recruitment of monocytes and granulocytes. It is known that members of the CC family target primarily monocytes and T cells, whereas CXC chemokines affect mainly neutrophils. It has been previously reported that the existence of different monocyte subsets expressing different chemokine receptors display distinct migratory and functional properties. Interestingly, the profile by MSCs in response to chemokines secreted IL-1β, was enriched in CCL5, CCL20 and CX3CL1, that could specifically attract not only neutrophils and monocytes, but also monocytes expressing CCR5, CCR6 and high levels of CX3CR1 [34]. Although leukocyte chemotaxis and lymphocyte development are the main functional properties of chemokines, they posses other biological activities like regulation of angiogenesis, control of cell proliferation and alteration of the expression of adhesion molecules. Indeed, the structural ERL domain present in several members of the CXC chemokine family determines their angiogenic potential [35] and the induced chemokquines CXCL1, CXCL3, and CXCL8 (IL-8) contain this motif. In the same context, CXCL10 is considered a “stop signal” that limits expansion of the fibrotic reaction triggered by TGFβ, FGF, and VEGF during myocardial healing [31]. The high levels of activation of this chemokine in MSC (Table 3) could account for the potent ability of these cells to control adverse remodeling during myocardial healing [8, 36, 37].

Claudins are transmembrane proteins found in tight juntions that participate not only in regulating tissue barrier function and permeability but also in cell motility, adhesion and migration [38]. Claudins (CLDN1 and CLDN14) were up-regulated in MSC after IL-1β treatment. A similar response has been reported in airway smooth muscle cells in response to IL-1β and TNFα [39], indicating similar activation pathways. It has been described that TLR signalling is linked to NF-κB and MAPK signalling pathways, and that this induction mediates the secretion chemokines and regulates immunosuppressive activity and recruitment of innate immune cells [21, 40, 41]. TLR2 and TLR4 were up-regulated in response to IL-1β. Similar effect had been previously described after stimulation with LPS of MSC from human parotid glands [42].

We also found differences between the activation pattern of MSC in response to different inflammatory mediators. Whereas TNFα increased preferentially CCL2 (MCP-1), CCL5 (RANTES), CXCL1, CXCL5, CXCL8, CXCL10 and CCL11 [10], we demonstrate here that IL-1β increases preferentially CCL3, CCL5, CCL20, CXCL1,CXCL3, CXCL10 and CXCL11. Thus, modulation of MSC biological responses is closely associated with culture conditions and the presence of immune mediators influence MSC proliferation and multipotency. In this context, culture protocols with milieu capable of MSC expansion while preserving chromosome stability have been developed [43]

In summary, our findings show that IL-1β increases migration and adhesion of MSC and promotes leucocyte chemotaxis through MSC secretion of soluble factors. As described in other cell types [44], IL-1β activates NF-κB resultings in transcriptional activation of a wide variety of genes such inflammatory mediators, adhesion molecules, growth factor or immune response mediator. Since some of these molecules are chemotactic for inflammatory leukocytes, like monocytes and neutrophils, these paracrine factors could facilitate infiltration of immune cells for tissue repair when MSC are transplanted into injured tissues.

Taken together, these findings shed light on MSC behaviour in inflammatory environments and suggest that inflammatory mediators like IL-1β induce a response in MSC that could trigger paracrine actions *in vivo*.

## Electronic supplementary material

Below is the link to the electronic supplementary material.

Table S1 Identification of genes from enriched biological processes up-regulated in MSC-IL1β. Fold changes were calculated between two experimental conditions as log 2 transformation of the ratio between MSC and MSC-IL1β. Systematic name, gene symbol and description of genes with significant changes are indicated. Minus values of fold change (Fc) indicate up-regulation in MSC-IL1β. (DOC 874 kb)

## References

[1] Pereira RF, O'Hara MD, Laptev AV, Halford KW, Pollard MD, Class R, Simon D, Livezey K, Prockop DJ (1998). Marrow stromal cells as a source of progenitor cells for nonhematopoietic tissues in transgenic mice with a phenotype of osteogenesis imperfecta. Proceedings of the National Academy of Sciences of the United States of America.

[2] Dezawa M, Ishikawa H, Itokazu Y, Yoshihara T, Hoshino M, Takeda S, Ide C, Nabeshima Y (2005). Bone marrow stromal cells generate muscle cells and repair muscle degeneration. Science.

[3] McFarlin K, Gao X, Liu YB, Dulchavsky DS, Kwon D, Arbab AS, Bansal M, Li Y, Chopp M, Dulchavsky SA, Gautam SC (2006). Bone marrow-derived mesenchymal stromal cells accelerate wound healing in the rat. Wound Repair and Regeneration.

[4] Chen L, Tredget EE, Wu PY, Wu Y (2008). Paracrine factors of mesenchymal stem cells recruit macrophages and endothelial lineage cells and enhance wound healing. PLoS One.

[5] Gnecchi M, He H, Noiseux N, Liang OD, Zhang L, Morello F, Mu H, Melo LG, Pratt RE, Ingwall JS, Dzau VJ (2006). Evidence supporting paracrine hypothesis for Akt-modified mesenchymal stem cell-mediated cardiac protection and functional improvement. The FASEB Journal.

[6] Kinnaird T, Stabile E, Burnett MS, Lee CW, Barr S, Fuchs S, Epstein SE (2004). Marrow-derived stromal cells express genes encoding a broad spectrum of arteriogenic cytokines and promote in vitro and in vivo arteriogenesis through paracrine mechanisms. Circulation Research.

[7] Uemura R, Xu M, Ahmad N, Ashraf M (2006). Bone marrow stem cells prevent left ventricular remodeling of ischemic heart through paracrine signaling. Circulation Research.

[8] Arminan A, Gandia C, Garcia-Verdugo JM, Lledo E, Trigueros C, Ruiz-Sauri A, Minana MD, Solves P, Paya R, Montero JA, Sepulveda P (2010). Mesenchymal stem cells provide better results than hematopoietic precursors for the treatment of myocardial infarction. Journal of the American College of Cardiology.

[9] Kawada H, Fujita J, Kinjo K, Matsuzaki Y, Tsuma M, Miyatake H, Muguruma Y, Tsuboi K, Itabashi Y, Ikeda Y, Ogawa S, Okano H, Hotta T, Ando K, Fukuda K (2004). Nonhematopoietic mesenchymal stem cells can be mobilized and differentiate into cardiomyocytes after myocardial infarction. Blood.

[10] Ponte AL, Marais E, Gallay N, Langonne A, Delorme B, Herault O, Charbord P, Domenech J (2007). The in vitro migration capacity of human bone marrow mesenchymal stem cells: comparison of chemokine and growth factor chemotactic activities. Stem Cells.

[11] Ren G, Zhang L, Zhao X, Xu G, Zhang Y, Roberts AI, Zhao RC, Shi Y (2008). Mesenchymal stem cell-mediated immunosuppression occurs via concerted action of chemokines and nitric oxide. Cell Stem Cell.

[12] Greco SJ, Rameshwar P (2007). Enhancing effect of IL-1alpha on neurogenesis from adult human mesenchymal stem cells: implication for inflammatory mediators in regenerative medicine. Journal of Immunology.

[13] Bednarski BK, Ding X, Coombe K, Baldwin AS, Kim HJ (2008). Active roles for inhibitory kappaB kinases alpha and beta in nuclear factor-kappaB-mediated chemoresistance to doxorubicin. Molecular Cancer Therapeutics.

[14] Bolstad BM, Irizarry RA, Astrand M, Speed TP (2003). A comparison of normalization methods for high density oligonucleotide array data based on variance and bias. Bioinformatics.

[15] Al-Shahrour F, Minguez P, Tarraga J, Medina I, Alloza E, Montaner D, Dopazo J (2007). FatiGO +: a functional profiling tool for genomic data. Integration of functional annotation, regulatory motifs and interaction data with microarray experiments. Nucleic Acids Research.

[16] Medina, I., Carbonell, J., Pulido, L., Madeira, S. C., Goetz, S., Conesa, A., Tarraga, J., Pascual-Montano, A., Nogales-Cadenas, R., Santoyo, J., Garcia, F., Marba, M., Montaner, D., & Dopazo, J. (2010). Babelomics: an integrative platform for the analysis of transcriptomics, proteomics and genomic data with advanced functional profiling. Nucleic Acids Res 38:W210-3. doi:10.1093/nar/gkq388 10.1093/nar/gkq388 PMC2896184 20478823

[17] Moser B, Loetscher P (2001). Lymphocyte traffic control by chemokines. Nature Immunology.

[18] Spanaus KS, Nadal D, Pfister HW, Seebach J, Widmer U, Frei K, Gloor S, Fontana A (1997). C-X-C and C-C chemokines are expressed in the cerebrospinal fluid in bacterial meningitis and mediate chemotactic activity on peripheral blood-derived polymorphonuclear and mononuclear cells in vitro. Journal of Immunology.

[19] Gordon, S., & Martinez, F. O. (2010). Alternative activation of macrophages: mechanism and functions. *Immunity, 32*, 593–604. 10.1016/j.immuni.2010.05.007 20510870

[20] Kim, H. S., Shin, T. H., Yang, S. R., Seo, M. S., Kim, D. J., Kang, S. K., Park, J. H., & Kang, K. S. Implication of NOD1 and NOD2 for the differentiation of multipotent mesenchymal stem cells derived from human umbilical cord blood. PLoS One 5:e15369. 10.1371/journal.pone.0015369 PMC2962653 21042538

[21] Tomchuck SL, Zwezdaryk KJ, Coffelt SB, Waterman RS, Danka ES, Scandurro AB (2008). Toll-like receptors on human mesenchymal stem cells drive their migration and immunomodulating responses. Stem Cells.

[22] Marui N, Offermann MK, Swerlick R, Kunsch C, Rosen CA, Ahmad M, Alexander RW, Medford RM (1993). Vascular cell adhesion molecule-1 (VCAM-1) gene transcription and expression are regulated through an antioxidant-sensitive mechanism in human vascular endothelial cells. The Journal of Clinical Investigation.

[23] Kim YS, Park HJ, Hong MH, Kang PM, Morgan JP, Jeong MH, Cho JG, Park JC, Ahn Y (2009). TNF-alpha enhances engraftment of mesenchymal stem cells into infarcted myocardium. Frontiers in Bioscience.

[24] Zhang D, Fan GC, Zhou X, Zhao T, Pasha Z, Xu M, Zhu Y, Ashraf M, Wang Y (2008). Over-expression of CXCR4 on mesenchymal stem cells augments myoangiogenesis in the infarcted myocardium. Journal of Molecular and Cellular Cardiology.

[25] Hayden MS, Ghosh S (2004). Signaling to NF-kappaB. Genes & Development.

[26] Mercurio F, Zhu H, Murray BW, Shevchenko A, Bennett BL, Li J, Young DB, Barbosa M, Mann M, Manning A, Rao A (1997). IKK-1 and IKK-2: cytokine-activated IkappaB kinases essential for NF-kappaB activation. Science.

[27] Baker, R. G., Hayden, M. S., & Ghosh, S. (2011). NF-kappaB, inflammation, and metabolic disease. *Cell Metab*, *13*, 11–22. 10.1016/j.cmet.2010.12.008 PMC3040418 21195345

[28] Aggarwal S, Pittenger MF (2005). Human mesenchymal stem cells modulate allogeneic immune cell responses. Blood.

[29] Di Nicola M, Carlo-Stella C, Magni M, Milanesi M, Longoni PD, Matteucci P, Grisanti S, Gianni AM (2002). Human bone marrow stromal cells suppress T-lymphocyte proliferation induced by cellular or nonspecific mitogenic stimuli. Blood.

[30] Raffaghello L, Bianchi G, Bertolotto M, Montecucco F, Busca A, Dallegri F, Ottonello L, Pistoia V (2008). Human mesenchymal stem cells inhibit neutrophil apoptosis: a model for neutrophil preservation in the bone marrow niche. Stem Cells.

[31] Bujak M, Frangogiannis NG (2009). The role of IL-1 in the pathogenesis of heart disease. Archivum Immunologiae et Therapiae Experimentalis.

[32] Donath, M. Y., & Shoelson, S. E. (2011). Type 2 diabetes as an inflammatory disease. *Nat Rev Immunol*, *11*, 98–107. 10.1038/nri2925 21233852

[33] Shaftel SS, Griffin WS, O'Banion MK (2008). The role of interleukin-1 in neuroinflammation and Alzheimer disease: an evolving perspective. Journal of Neuroinflammation.

[34] Tacke F, Alvarez D, Kaplan TJ, Jakubzick C, Spanbroek R, Llodra J, Garin A, Liu J, Mack M, van Rooijen N, Lira SA, Habenicht AJ, Randolph GJ (2007). Monocyte subsets differentially employ CCR2, CCR5, and CX3CR1 to accumulate within atherosclerotic plaques. The Journal of Clinical Investigation.

[35] Le Y, Zhou Y, Iribarren P, Wang J (2004). Chemokines and chemokine receptors: their manifold roles in homeostasis and disease. Cellular and molecular immunology.

[36] Amado LC, Saliaris AP, Schuleri KH, St John M, Xie JS, Cattaneo S, Durand DJ, Fitton T, Kuang JQ, Stewart G, Lehrke S, Baumgartner WW, Martin BJ, Heldman AW, Hare JM (2005). Cardiac repair with intramyocardial injection of allogeneic mesenchymal stem cells after myocardial infarction. Proceedings of the National Academy of Sciences of the United States of America.

[37] Davani S, Marandin A, Mersin N, Royer B, Kantelip B, Herve P, Etievent JP, Kantelip JP (2003). Mesenchymal progenitor cells differentiate into an endothelial phenotype, enhance vascular density, and improve heart function in a rat cellular cardiomyoplasty model. Circulation.

[38] Leotlela PD, Wade MS, Duray PH, Rhode MJ, Brown HF, Rosenthal DT, Dissanayake SK, Earley R, Indig FE, Nickoloff BJ, Taub DD, Kallioniemi OP, Meltzer P, Morin PJ, Weeraratna AT (2007). Claudin-1 overexpression in melanoma is regulated by PKC and contributes to melanoma cell motility. Oncogene.

[39] Fujita, H., Chalubinski, M., Rhyner, C., Indermitte, P., Meyer, N., Ferstl, R., Treis, A., Gomez, E., Akkaya, A., O'Mahony, L., Akdis, M., & Akdis, C. A. (2011). Claudin-1 expression in airway smooth muscle exacerbates airway remodeling in asthmatic subjects. *J Allergy Clin Immunol*, *127*, 1612–21 e8. 10.1016/j.jaci.2011.03.039 21624620

[40] Liotta F, Angeli R, Cosmi L, Fili L, Manuelli C, Frosali F, Mazzinghi B, Maggi L, Pasini A, Lisi V, Santarlasci V, Consoloni L, Angelotti ML, Romagnani P, Parronchi P, Krampera M, Maggi E, Romagnani S, Annunziato F (2008). Toll-like receptors 3 and 4 are expressed by human bone marrow-derived mesenchymal stem cells and can inhibit their T-cell modulatory activity by impairing Notch signaling. Stem Cells.

[41] Romieu-Mourez R, Francois M, Boivin MN, Bouchentouf M, Spaner DE, Galipeau J (2009). Cytokine modulation of TLR expression and activation in mesenchymal stromal cells leads to a proinflammatory phenotype. Journal of Immunology.

[42] Brandau, S., Jakob, M., Hemeda, H., Bruderek, K., Janeschik, S., Bootz, F., & Lang, S. (2010). Tissue-resident mesenchymal stem cells attract peripheral blood neutrophils and enhance their inflammatory activity in response to microbial challenge. *J Leukoc Biol*, *88*, 1005–15. 10.1189/jlb.0410207 20682625

[43] Crespo-Diaz R, Behfar A, Butler GW, Padley DJ, Sarr MG, Bartunek J, Dietz AB, Terzic A (2011). Platelet lysate consisting of a natural repair proteome supports human mesenchymal stem cell proliferation and chromosomal stability. Cell Transplantation.

[44] Collins T, Read MA, Neish AS, Whitley MZ, Thanos D, Maniatis T (1995). Transcriptional regulation of endothelial cell adhesion molecules: NF-kappa B and cytokine-inducible enhancers. The FASEB Journal.

