# Supplementary material for: IL1β Induces Mesenchymal Stem Cells Migration and Leucocyte Chemotaxis Through NF-κB
Source: Stem Cell Rev. 2012 Mar 31;8(3):905–16. doi: 10.1007/s12015-012-9364-9 (PMC3412085; doi:10.1007/s12015-012-9364-9)
Supplement: Supplementary file 1 — Identification of genes from enriched biological processes up-regulated in MSC-IL1β. Fold changes were calculated between two experimental conditions as log 2 transformation of the ratio between MSC and MSC-IL1β. Systematic name, gene symbol and description of genes with significant changes are indicated. Minus values of fold change (Fc) indicate up-regulation in MSC-IL1β. (DOC 874 kb) [file 12015_2012_9364_MOESM1_ESM.doc]

Supplemental Table 1. Identification of genes from enriched biological processes up-regulated in MSC-IL1β. Fold changes were calculated between two experimental conditions as log 2 transformation of the ratio between MSC and MSC-IL1β. Systematic name, gene symbol and description of genes with significant changes are indicated. Minus values of fold change (Fc) indicate up-regulation in MSC-IL1β.

| **Blood Coagulation (GO:0007596)** | |  |  |  |
| --- | --- | --- | --- | --- |
| **Systematic Name** | **Probe Name** | **Gene Symbol** | **Description** | **Fc** |
| NM_001993 | A_23_P126782 | F3 | Coagulation factor III | -1.32 |
| AF118092 | A_24_P359491 | AF118092 | PRO2061 | -0.96 |
| ENST00000248076 | A_24_P228470 | PAR4 | Protease-activated receptor 4 | -0.83 |
| ENST00000222543 | A_24_P95070 |  | cDNA FLJ26323 fis | -0.76 |
| NM_000675 | A_23_P109436 | ADORA2A | Adenosine A2a receptor | -0.76 |
| J02940 | A_23_P152926 | GP1BA | Platelet glycoprotein Ib alpha chain | -0.69 |
| NM_005242 | A_23_P58835 | F2RL1 | Coagulation factor II | -0.55 |
| NM_000641 | A_23_P67169 | IL11 | Interleukin 11 | -0.34 |
| NM_000312 | A_23_P40096 | PROC | Protein C | -0.32 |
| NM_000602 | A_24_P158089 | SERPINE1 | Serpin peptidase inhibitor | -0.30 |
| NM_000488 | A_23_P114626 | SERPINC1 | Serpin peptidase inhibitor | -0.27 |
| NM_000361 | A_23_P91390 | THBD | Thrombomodulin | -0.27 |
| NM_006528 | A_23_P393620 | TFPI2 | Tissue factor pathway inhibitor 2 | -0.22 |
| NM_000212 | A_23_P38519 | ITGB3 | Integrin-beta 3 | -0.21 |
|  |  |  |  |  |
| **Cell Adhesion (GO:0007155)** | |  |  |  |
| **Systematic Name** | **Probe Name** | **Gene Symbol** | **Description** | **Fc** |
| NM_000584 | A_32_P87013 | IL8 | Interleukin 8 | -2.61 |
| NM_000450 | A_23_P97112 | SELE | Selectin E | -1.67 |
| NM_002985 | A_23_P152838 | CCL5 | Chemokine (C-C motif) ligand 5 | -1.34 |
| NM_021101 | A_23_P57784 | CLDN1 | Claudin 1 | -1.12 |
| NM_004967 | A_23_P144549 | IBSP | Integrin-binding sialoprotein | -0.97 |
| NM_144492 | A_23_P91512 | CLDN14 | Claudin 14 | -0.95 |
| NM_000594 | A_23_P376488 | TNF | Tumor necrosis factor | -0.93 |
| NM_002996 | A_24_P381901 | CX3CL1 | Chemokine (C-X3-C motif) | -0.87 |
| NM_003734 | A_23_P426305 | AOC3 | Amine oxidase | -0.83 |
| NM_000201 | A_23_P153320 | ICAM1 | Intercellular adhesion molecule 1 | -0.81 |
| J02940 | A_23_P152926 | GP1BA | Platelet glycoprotein Ib alpha chain | -0.69 |
| NM_004062 | A_23_P100240 | CDH16 | Cadherin 16 KSP-cadherin | -0.68 |
| NM_018534 | A_23_P429555 | NRP2 | Neuropilin 2 | -0.66 |
| NM_000425 | A_24_P207995 | L1CAM | L1 cell adhesion molecule | -0.66 |
| NM_002982 | A_23_P89431 | CCL2 | Chemokine (C-C motif) ligand 2 | -0.60 |
| NM_016369 | A_23_P336693 | CLDN18 | Claudin 18 | -0.59 |
| NM_023068 | A_23_P17481 | SIGLEC1 | Sialic acid binding Ig-like lectin 1 | -0.53 |
| NM_001078 | A_23_P34345 | VCAM1 | Vascular cell adhesion molecule 1 | -0.53 |
| NM_013280 | A_23_P47168 | FLRT1 | Fibronectin leucine rich transmembrane protein 1 | -0.52 |
| NM_001523 | A_23_P27400 | HAS1 | Hyaluronan synthase 1 | -0.51 |
| NM_007115 | A_23_P165624 | TNFAIP6 | TNF alpha-induced protein 6 | -0.50 |
| NM_014470 | A_23_P53370 | RND1 | Rho family GTPase 1 | -0.50 |
| NM_201266 | A_23_P209669 | NRP2 | Neuropilin 2 | -0.45 |
| NM_004613 | A_32_P86763 | TGM2 | Transglutaminase 2 | -0.40 |
| NM_138938 | A_23_P119936 | REG3A | Regenerating islet-derived 3 alpha | -0.39 |
| NM_002589 | A_23_P310921 | PCDH7 | BH-protocadherin | -0.38 |
| NM_002986 | A_23_P66635 | CCL11 | Chemokine (C-C motif) ligand 11 | -0.38 |
| NM_033100 | A_23_P149946 | PCDH21 | Protocadherin 21 | -0.37 |
| NM_005725 | A_23_P201193 | TSPAN2 | Tetraspanin 2 | -0.36 |
| NM_006403 | A_23_P344555 | NEDD9 | Neural precursor cell expressed | -0.35 |
| NM_003152 | A_23_P207367 | STAT5A | Signal transducer and activator of transcription 5A | -0.32 |
| NM_000346 | A_23_P26847 | SOX9 | SRY (sex determining region Y)-box 9 | -0.31 |
| NM_002984 | A_23_P207564 | CCL4 | Chemokine (C-C motif) ligand 4 | -0.30 |
| AK074780 | A_24_P376150 | ROBO2 | cDNA FLJ90299 fis, clone NT2RP2000514 | -0.29 |
| AF193046 | A_24_P921436 | FBN2 | PP187 | -0.29 |
| NM_001305 | A_24_P115183 | CLDN4 | Claudin 4 | -0.28 |
| NM_018891 | A_23_P160968 | LAMC2 | Laminin. gamma 2 | -0.27 |
| NM_001203 | A_24_P63380 | BMPR1B | Bone morphogenetic protein receptor type IB | -0.27 |
| NM_004148 | A_23_P169137 | NINJ1 | Ninjurin 1 | -0.26 |
| NM_006727 | A_23_P144656 | CDH10 | Cadherin 10 type 2 (T2-cadherin) | -0.25 |
| NM_005353 | A_23_P129665 | ITGAD | Integrin-alpha D | -0.25 |
| NM_181861 | A_23_P36611 | APAF1 | Apoptotic peptidase activating factor | -0.23 |
| NM_201264 | A_24_P50801 | NRP2 | Neuropilin 2 | -0.23 |
| NM_016242 | A_23_P382065 | EMCN | Endomucin | -0.22 |
| NM_181847 | A_23_P14083 | AMIGO2 | Adhesion molecule with Ig-like domain 2 | -0.21 |
| NM_003277 | A_23_P6321 | CLDN5 | Claudin 5 | -0.21 |
| NM_020815 | A_23_P170238 | PCDH10 | Protocadherin 10 | -0.21 |
| NM_000212 | A_23_P38519 | ITGB3 | Integrin. beta 3 | -0.21 |
| NM_001544 | A_23_P130537 | ICAM4 | Intercellular adhesion molecule 4 | -0.21 |
| NM_080792 | A_23_P210708 | SIRPA | Signal-regulatory protein alpha | -0.20 |
| NM_000330 | A_23_P148292 | RS1 | Retinoschisis (X-linked. juvenile) 1 | -0.20 |
| A_24_P927917 | A_24_P927917 | A_24_P927917 | Unknown | -0.20 |
| NM_005562 | A_23_P201636 | LAMC2 | Laminin gamma 2 | -0.19 |
| NM_006769 | A_23_P380181 | LMO4 | LIM domain only 4 | -0.18 |
| NM_005201 | A_23_P211699 | CCR8 | Chemokine (C-C motif) receptor 8 | -0.18 |
| AJ008005 | A_23_P106174 | PSEN1 | PSN1 gene. alternative transcript | -0.18 |
| BC002828 | A_24_P256513 | AGGF1 | Angiogenic factor with G patch and FHA domains 1 | -0.17 |
| NM_003259 | A_23_P119143 | ICAM5 | Intercellular adhesion molecule 5 | -0.17 |
| NM_198951 | A_24_P923251 | TGM2 | Transglutaminase 2 | -0.17 |
| NM_152750 | A_23_P348253 | FLJ23834 | Hypothetical protein FLJ23834 | -0.15 |
| NM_014718 | A_23_P53724 | CLSTN3 | Calsyntenin 3 | -0.15 |
| NM_001772 | A_24_P301655 | CD33 | CD33 molecule | -0.15 |
| NM_005985 | A_23_P131846 | SNAI1 | Snail homolog 1 | -0.15 |
| NM_152733 | A_32_P113566 | BTBD9 | BTB (POZ) domain containing 9 | -0.15 |
| A_32_P1076 | A_32_P1076 | A_32_P1076 | Unknown | -0.15 |
| NM_013994 | A_24_P123601 | DDR1 | Discoidin domain receptor family. member 1 | -0.14 |
| NM_004572 | A_23_P162466 | PKP2 | Plakophilin 2 | -0.14 |
| NM_019863 | A_23_P217643 | F8 | Coagulation factor VIII | -0.14 |
| NM_014751 | A_23_P347632 | MTSS1 | Metastasis suppressor 1 | -0.14 |
| NM_021155 | A_24_P305345 | CD209 | CD209 molecule | -0.14 |
| NM_012129 | A_23_P157268 | CLDN12 | Claudin 12 | -0.13 |
| NM_138931 | A_23_P57856 | BCL6 | B-cell CLL/lymphoma 6 | -0.13 |
| XM_942822 | A_32_P703 | LOC646626 | Hypothetical protein LOC647393 | -0.13 |
| NM_003506 | A_23_P123276 | FZD6 | Frizzled homolog 6 | -0.13 |
| NM_001943 | A_23_P141730 | DSG2 | Desmoglein 2 | -0.13 |
| NM_004407 | A_23_P133153 | DMP1 | Dentin matrix acidic phosphoprotein | -0.13 |
| NM_000632 | A_23_P124108 | ITGAM | Integrin. alpha M | -0.13 |
| NM_020882 | A_32_P185637 | COL20A1 | Collagen. type XX alpha 1 | -0.13 |
| NM_198148 | A_23_P138524 | CPXM2 | Carboxypeptidase X | -0.13 |
| A_32_P215745 | A_32_P215745 | A_32_P215745 | Unknown | -0.13 |
| NM_002716 | A_24_P98762 | PPP2R1B | Protein phosphatase 2 | -0.12 |
| NM_002214 | A_24_P273599 | ITGB8 | Integrin beta 8 | -0.12 |
| NM_014442 | A_24_P40001 | SIGLEC8 | Sialic acid binding Ig-like lectin 8 | -0.12 |
| NM_007314 | A_23_P138099 | ABL2 | V-abl Abelson murine leukemia viral oncogene homolog 2 | -0.12 |
| NM_005157 | A_24_P281101 | ABL1 | V-abl Abelson murine leukemia viral oncogene homolog 1 | -0.12 |
| NM_005168 | A_23_P142849 | RND3 | Rho family GTPase 3 | -0.11 |
| NM_014479 | A_23_P256425 | ADAMDEC1 | ADAM-like. decysin 1 | -0.11 |
| NM_001779 | A_23_P138308 | CD58 | CD58 molecule | -0.11 |
| BC020868 | A_24_P342178 | STAT5B | Signal transducer and activator of transcription 5B | -0.11 |
| NM_006725 | A_23_P139162 | CD6 | CD6 molecule | -0.10 |
| NM_024690 | A_23_P5211 | MUC16 | Mucin 16. cell surface associated | -0.10 |
| NM_001337 | A_23_P407565 | CX3CR1 | Chemokine (C-X3-C motif) receptor 1 | -0.10 |
| NM_001663 | A_23_P48691 | ARF6 | ADP-ribosylation factor 6 | -0.10 |
| NM_003830 | A_23_P15995 | SIGLEC5 | Sialic acid binding Ig-like lectin 5 | -0.10 |
| NM_005245 | A_23_P69586 | FAT | FAT tumor suppressor homolog 1 | -0.09 |
| NM_004762 | A_23_P83781 | PSCD1 | Pleckstrin homology. Sec7 and coiled-coil domains 1 | -0.09 |
| NM_016639 | A_23_P49338 | TNFRSF12A | Tumor necrosis factor receptor superfamily | -0.09 |
| NM_152403 | A_23_P144911 | FLJ39155 | Hypothetical protein FLJ39155 | -0.09 |
| NM_002160 | A_23_P157865 | TNC | Tenascin C | -0.09 |
| NM_003921 | A_23_P115286 | BCL10 | B-cell CLL/lymphoma 10 | -0.09 |
| NM_173670 | A_24_P363100 | RGMB | RGM domain family. member B | -0.09 |
| NM_004386 | A_23_P153797 | CSPG3 | Chondroitin sulfate proteoglycan 3 | -0.09 |
| NM_012448 | A_23_P100788 | STAT5B | Signal transducer and activator of transcription 5B | -0.08 |
| NM_012192 | A_23_P127851 | FXC1 | Fracture callus 1 homolog | -0.08 |
| NM_021181 | A_24_P353638 | SLAMF7 | SLAM family member 7 | -0.08 |
| NM_145351 | A_23_P15414 | SCARF1 | Scavenger receptor class F. member 1 | -0.08 |
| NM_152888 | A_32_P405759 | COL22A1 | Collagen. type XXII alpha 1 | -0.07 |
| NM_148960 | A_23_P379054 | CLDN19 | Claudin 19 | -0.07 |
| NM_005228 | A_23_P215790 | EGFR | Epidermal growth factor receptor | -0.07 |
| NM_003285 | A_23_P45864 | TNR | Tenascin R | -0.07 |
| NM_016580 | A_23_P423309 | PCDH12 | Protocadherin 12 | -0.07 |
| NM_021219 | A_23_P120667 | JAM2 | Junctional adhesion molecule 2 | -0.07 |
| NM_004040 | A_23_P51136 | RHOB | Ras homolog gene family member B | -0.07 |
| NM_001400 | A_23_P404481 | EDG1 | Endothelial differentiation | -0.07 |
| NM_016952 | A_23_P98335 | CDON | Cdon homolog | -0.07 |
| NM_006149 | A_23_P254917 | LGALS4 | Lectin. galactoside-binding soluble 4 | -0.07 |
| NM_003882 | A_23_P354694 | WISP1 | WNT1 inducible signaling pathway protein 1 | -0.06 |
| NM_016184 | A_23_P48029 | CLEC4A | C-type lectin domain family 4 member A | -0.06 |
| NM_000494 | A_23_P52323 | COL17A1 | Collagen. type XVII Alpha 1 | -0.06 |
| NM_032011 | A_24_P334529 | PCDHGA3 | Protocadherin gamma subfamily A3 | -0.06 |
| NM_007261 | A_23_P207037 | CD300A | CD300a molecule | -0.06 |
| NM_024814 | A_23_P146004 | CBLL1 | Cas-Br-M ecotropic retroviral transforming sequence-like 1 | -0.06 |
| NM_018935 | A_23_P121851 | PCDHB15 | Protocadherin beta 15 | -0.06 |
| NM_002855 | A_24_P50890 | PVRL1 | Poliovirus receptor-related 1 | -0.06 |
|  |  |  |  |  |
| **Chemotaxis (GO:0006935)** | |  |  |  |
| **Systematic Name** | **Probe Name** | **Gene Symbol** | **Description** | **Fc** |
| NM_000584 | A_32_P87013 | IL8 | Interleukin 8 | -2.61 |
| NM_001565 | A_24_P303091 | CXCL10 | Chemokine (C-X-C motif) ligand 10 | -2.26 |
| NM_004591 | A_23_P17065 | CCL20 | Chemokine (C-C motif) ligand 20 | -2.22 |
| NM_000576 | A_23_P79518 | IL1B | Interleukin 1beta | -2.13 |
| NM_000575 | A_23_P72096 | IL1A | Interleukin 1alpha | -1.62 |
| NM_002993 | A_23_P155755 | CXCL6 | Chemokine (C-X-C motif) ligand 6 | -1.59 |
| NM_005623 | A_23_P207456 | CCL8 | Chemokine (C-C motif) ligand 8 | -1.57 |
| NM_002985 | A_23_P152838 | CCL5 | Chemokine (C-C motif) ligand 5 | -1.34 |
| NM_002089 | A_23_P315364 | CXCL2 | Chemokine (C-X-C motif) ligand 2 | -1.32 |
| NM_006273 | A_23_P78037 | CCL7 | Chemokine (C-C motif) ligand 7 | -1.26 |
| NM_005409 | A_24_P20607 | CXCL11 | Chemokine (C-X-C motif) ligand 11 | -1.11 |
| NM_001511 | A_23_P7144 | CXCL1 | Chemokine (C-X-C motif) ligand 1 | -1.00 |
| NM_002090 | A_24_P183150 | CXCL3 | Chemokine (C-X-C motif) ligand 3 | -0.97 |
|  |  |  |  |  |
| **Defense Response (GO:0006952)** | |  |  |  |
| **Systematic Name** | **Probe Name** | **Gene Symbol** | **Description** | **Fc** |
| NM_000584 | A_32_P87013 | IL8 | Interleukin 8 | -2.61 |
| NM_000758 | A_23_P133408 | CSF2 | Colony stimulating factor 2 | -2.27 |
| NM_001565 | A_24_P303091 | CXCL10 | Chemokine (C-X-C motif) ligand 10 | -2.26 |
| NM_004591 | A_23_P17065 | CCL20 | Chemokine (C-C motif) ligand 20 | -2.22 |
| NM_000576 | A_23_P79518 | IL1B | Interleukin 1beta | -2.13 |
| NM_022162 | A_23_P420863 | CARD15 | Caspase recruitment domain family. member 15 | -1.69 |
| NM_000450 | A_23_P97112 | SELE | Selectin E | -1.67 |
| NM_000575 | A_23_P72096 | IL1A | Interleukin 1alpha | -1.62 |
| NM_002993 | A_23_P155755 | CXCL6 | Chemokine (C-X-C motif) ligand 6 | -1.59 |
| NM_005623 | A_23_P207456 | CCL8 | Chemokine (C-C motif) ligand 8 | -1.57 |
| NM_000759 | A_23_P501754 | CSF3 | Colony stimulating factor 3 | -1.41 |
| NM_003785 | A_24_P314337 | PAGE1 | P antigen family member 1 | -1.37 |
| NM_002985 | A_23_P152838 | CCL5 | Chemokine (C-C motif) ligand 5 | -1.34 |
| NM_002089 | A_23_P315364 | CXCL2 | Chemokine (C-X-C motif) ligand 2 | -1.32 |
| NM_001432 | A_23_P41344 | EREG | Epiregulin | -1.32 |
| NM_006273 | A_23_P78037 | CCL7 | Chemokine (C-C motif) ligand 7 | -1.26 |
| NM_030956 | A_23_P33420 | TLR10 | Toll-like receptor 10 | -1.17 |
| NM_003264 | A_23_P92499 | TLR2 | Toll-like receptor 2 | -1.12 |
| NM_005409 | A_24_P20607 | CXCL11 | Chemokine (C-X-C motif) ligand 11 | -1.11 |
| NM_014589 | A_23_P114857 | PLA2G2E | Phospholipase A2 group IIE | -1.08 |
| NM_001511 | A_23_P7144 | CXCL1 | Chemokine (C-X-C motif) ligand 1 | -1.00 |
| NM_002090 | A_24_P183150 | CXCL3 | Chemokine (C-X-C motif) ligand 3 | -0.97 |
| NM_000594 | A_23_P376488 | TNF | Tumor necrosis factor | -0.93 |
| NM_001570 | A_23_P80635 | IRAK2 | Interleukin-1 receptor-associated kinase 2 | -0.90 |
| NM_002176 | A_23_P71774 | IFNB1 | Interferon. beta 1. fibroblast | -0.90 |
| NM_000963 | A_24_P250922 | PTGS2 | Prostaglandin-endoperoxide synthase 2 | -0.88 |
| NM_002996 | A_24_P381901 | CX3CL1 | Chemokine (C-X3-C motif) ligand 1 | -0.87 |
| NM_003734 | A_23_P426305 | AOC3 | Amine oxidase. copper containing 3 | -0.83 |
| NM_000600 | A_23_P71037 | IL6 | Interleukin 6 | -0.79 |
| NM_005894 | A_23_P126584 | CD5L | CD5 molecule-like | -0.78 |
| NM_000675 | A_23_P109436 | ADORA2A | Adenosine A2a receptor | -0.76 |
| NM_002983 | A_23_P373017 | CCL3 | Chemokine (C-C motif) ligand 3 | -0.76 |
| NM_004233 | A_23_P70670 | CD83 | CD83 molecule | -0.76 |
| NM_003182 | A_23_P215283 | TAC1 | Tachykinin. precursor 1 | -0.70 |
| NM_006737 | A_23_P101636 | KIR3DL2 | Killer cell immunoglobulin-like receptor | -0.70 |
| NM_001200 | A_23_P143331 | BMP2 | Bone morphogenetic protein 2 | -0.60 |
| NM_002982 | A_23_P89431 | CCL2 | Chemokine (C-C motif) ligand 2 | -0.60 |
| NM_016584 | A_23_P76078 | IL23A | Interleukin 23 alpha subunit p19 | -0.58 |
| NM_002704 | A_23_P121596 | PPBP | Pro-platelet basic protein | -0.55 |
| NM_001001437 | A_23_P321920 | CCL3L3 | Chemokine (C-C motif) ligand 3-like 3 | -0.53 |
| NM_023068 | A_23_P17481 | SIGLEC1 | Sialic acid binding Ig-like lectin 1 | -0.53 |
| NM_172140 | A_23_P337800 | IL29 | Interleukin 29 | -0.53 |
| NM_007115 | A_23_P165624 | TNFAIP6 | Tumor necrosis factor | -0.50 |
| NM_002994 | A_23_P110204 | CXCL5 | Chemokine (C-X-C motif) ligand 5 | -0.48 |
| NM_004843 | A_23_P27606 | IL27RA | Interleukin 27 receptor | -0.45 |
| NM_080869 | A_23_P68436 | WFDC12 | WAP four-disulfide core domain 12 | -0.42 |
| NM_004244 | A_23_P33723 | CD163 | CD163 molecule | -0.41 |
| NM_138938 | A_23_P119936 | REG3A | Regenerating islet-derived 3 alpha | -0.39 |
| NM_001066 | A_24_P54174 | TNFRSF1B | Tumor necrosis factor receptor superfamily | -0.39 |
| NM_058176 | A_23_P215577 | HDAC9 | Histone deacetylase 9 | -0.39 |
| NM_001805 | A_23_P2990 | CEBPE | CCAAT/enhancer binding protein | -0.38 |
| NM_000397 | A_23_P217258 | CYBB | Cytochrome b-245 beta polypeptide | -0.38 |
| NM_014707 | A_23_P404162 | HDAC9 | Histone deacetylase 9 | -0.38 |
| NM_000710 | A_23_P128744 | BDKRB1 | Bradykinin receptor B1 | -0.38 |
| NM_002986 | A_23_P66635 | CCL11 | Chemokine (C-C motif) ligand 11 | -0.38 |
| NM_000064 | A_23_P101407 | C3 | Complement component 3 | -0.37 |
| NM_005408 | A_24_P125335 | CCL13 | Chemokine (C-C motif) ligand 13 | -0.35 |
| NM_176891 | A_23_P302060 | IFNE1 | Interferon epsilon 1 | -0.35 |
| NM_003998 | A_23_P30024 | NFKB1 | Nuclear factor of kappa B-cells 1 | -0.35 |
|  |  |  |  |  |
| **I-kappaB kinase_NF-kappaB cascade (GO:0007249)** | | |  |  |
| **Systematic Name** | **Probe Name** | **Gene Symbol** | **Description** | **Fc** |
| NM_022162 | A_23_P420863 | CARD15 | Caspase recruitment domain family | -1.69 |
| NM_003807 | A_24_P237036 | TNFSF14 | Tumor necrosis factor (ligand) superfamily | -1.06 |
| NM_000594 | A_23_P376488 | TNF | Tumor necrosis factor | -0.93 |
| NM_001570 | A_23_P80635 | IRAK2 | Interleukin-1 receptor-associated kinase 2 | -0.90 |
| NM_006290 | A_24_P157926 | TNFAIP3 | Tumor necrosis factor. alpha-induced protein 3 | -0.85 |
| NM_002908 | A_23_P56938 | REL | V-rel reticuloendotheliosis viral oncogene homolog | -0.59 |
| NM_020529 | A_23_P106002 | NFKBIA | Nuclear factor of kappa B-cells inhibitor | -0.37 |
| NM_003821 | A_23_P252106 | RIPK2 | Receptor-interacting serine-threonine kinase 2 | -0.30 |
| AF009616 | A_23_P209394 | CFLAR | FLAME-1 | -0.29 |
| NM_000572 | A_23_P126735 | IL10 | Interleukin 10 | -0.25 |
| NM_005178 | A_23_P4662 | BCL3 | B-cell CLL/lymphoma 3 | -0.25 |
| NM_003879 | A_24_P120115 | CFLAR | CASP8 and FADD-like apoptosis regulator | -0.23 |
| NM_006622 | A_23_P30254 | PLK2 | Polo-like kinase 2 | -0.19 |
| NM_001250 | A_23_P57036 | CD40 | CD40 molecule TNF receptor superfamily | -0.17 |
| NM_004862 | A_23_P3532 | LITAF | Lipopolysaccharide-induced TNF factor | -0.16 |
| NM_014002 | A_23_P887 | IKBKE | Inhibitor of kappa B-cells | -0.15 |
| NM_001278 | A_23_P46748 | CHUK | Conserved helix-loop-helix ubiquitous kinase | -0.15 |
| NM_004310 | A_23_P58132 | RHOH | Ras homolog gene family member H | -0.15 |
| NM_182919 | A_23_P90311 | TICAM1 | Toll-like receptor adaptor molecule 1 | -0.14 |
| NM_021138 | A_23_P169331 | TRAF2 | TNF receptor-associated factor 2 | -0.13 |
| XM_942822 | A_32_P703 | LOC646626 | Hypothetical protein LOC647393 | -0.13 |
| NM_003842 | A_24_P218265 | TNFRSF10B | Tumor necrosis factor receptor superfamily | -0.13 |
| NM_003804 | A_23_P370005 | RIPK1 | Receptor (TNFRSF)-interacting serine-threonine kinase 1 | -0.12 |
| NM_001166 | A_24_P115774 | BIRC2 | Baculoviral IAP repeat-containing 2 | -0.12 |
| BC014095 | A_23_P104689 | RELA | V-rel reticuloendotheliosis viral oncogene homolog A | -0.11 |
| NM_003921 | A_23_P115286 | BCL10 | B-cell CLL/lymphoma 10 | -0.09 |
| NM_054014 | A_23_P397238 | FKBP1A | FK506 binding protein 1A. 12kDa | -0.06 |
| NM_016072 | A_23_P162425 | GOLT1B | Golgi transport 1 homolog B | -0.06 |
| NM_015093 | A_23_P19702 | MAP3K7IP2 | Mitogen-activated protein kinase | -0.06 |
| NM_003900 | A_23_P81399 | SQSTM1 | Sequestosome 1 | -0.06 |
| NM_133484 | A_24_P257108 | TANK | TRAF family member-associated NFKB activator | -0.05 |
| NM_000639 | A_23_P369815 | FASLG | Fas ligand | -0.05 |
| NM_153719 | A_24_P322444 | NUP62 | Nucleoporin 62kDa | -0.05 |
| NM_020205 | A_23_P138157 | OTUD7B | OTU domain containing 7B | -0.05 |
| NM_145803 | A_23_P75921 | TRAF6 | TNF receptor-associated factor 6 | -0.05 |
| NM_016463 | A_24_P930062 | CXXC5 | CXXC finger 5 | -0.05 |
| NM_014397 | A_23_P216920 | NEK6 | NIMA (never in mitosis gene a)-related kinase 6 | -0.04 |
| NM_057159 | A_23_P502879 | EDG2 | Endothelial differentiation | -0.04 |
| NM_003348 | A_23_P116829 | UBE2N | Ubiquitin-conjugating enzyme E2N | -0.04 |
| NM_138554 | A_23_P60306 | TLR4 | Toll-like receptor 4 | -0.04 |
| NM_138793 | A_23_P26759 | CANT1 | Calcium activated nucleotidase 1 | -0.04 |
| NM_032415 | A_23_P82324 | CARD11 | Caspase recruitment domain family | -0.04 |
| NM_006355 | A_23_P93236 | TRIM38 | Tripartite motif-containing 38 | -0.04 |
| NM_005415 | A_23_P165657 | SLC20A1 | Solute carrier family 20 (phosphate transporter) | -0.04 |
| NM_006070 | A_23_P29517 | TFG | TRK-fused gene | -0.03 |
| NM_004180 | A_23_P154306 | TANK | TRAF family member-associated NFKB activator | -0.03 |
| NM_014550 | A_23_P434890 | CARD10 | Caspase recruitment domain family | -0.03 |
| NM_019028 | A_23_P13065 | ZDHHC13 | Zinc finger DHHC-type containing 13 | -0.02 |
| AK090431 | A_23_P340019 | NOD3 |  | -0.02 |
| NM_016334 | A_23_P1056 | GPR89A | G protein-coupled receptor 89A | -0.02 |
| NM_003824 | A_24_P278637 | FADD | Fas (TNFRSF6)-associated via death domain | -0.02 |
| NM_002468 | A_23_P362659 | MYD88 | Myeloid differentiation primary response gene | -0.02 |
| A_24_P375932 | A_24_P375932 | A_24_P375932 | Unknown | -0.02 |
| NM_007315 | A_23_P56630 | STAT1 | Signal transducer and activator of transcription 1 | -0.02 |
| NM_178844 | A_23_P329399 | NOD3 | NOD3 protein | -0.02 |
| AK074557 | A_24_P297827 | TMED4 | cDNA FLJ90076 fis | -0.02 |
| NM_020644 | A_23_P162087 | TMEM9B | TMEM9 domain family | -0.01 |
| NM_203505 | A_24_P128977 | G3BP2 | Ras-GTPase activating protein SH3 domain-binding | -0.01 |
| NM_004619 | A_23_P201731 | TRAF5 | TNF receptor-associated factor 5 | -0.01 |
| NM_024544 | A_23_P200598 | C1orf166 | Chromosome 1 open reading frame 166 | -0.01 |
| NM_003265 | A_23_P29922 | TLR3 | Toll-like receptor 3 | -0.01 |
| NM_002133 | A_23_P120883 | HMOX1 | Heme oxygenase | -0.01 |
| A_32_P148636 | A_32_P148636 | A_32_P148636 | Unknown | -0.01 |
| NM_021649 | A_32_P123088 | TICAM2 | Toll-like receptor adaptor molecule 2 | -0.01 |
| NM_024110 | A_23_P207879 | CARD14 | Caspase recruitment domain family member 14 | -0.01 |
| NM_014959 | A_24_P14260 | CARD8 | Caspase recruitment domain family. member 8 | -0.01 |
| NM_004797 | A_23_P369237 | ADIPOQ | Adiponectin C1Q and collagen domain containing | -0.01 |
| NM_019080 | A_24_P264909 | NDFIP2 | Nedd4 family interacting protein 2 | -0.01 |
| NM_000165 | A_23_P93591 | GJA1 | Gap junction protein. alpha 1 | 0.01 |
| ENST00000379563 | A_23_P68539 |  | cDNA FLJ41962 | 0.01 |
| NM_001065 | A_23_P139722 | TNFRSF1A | Tumor necrosis factor receptor superfamily | 0.01 |
| NM_000061 | A_23_P137139 | BTK | Bruton agammaglobulinemia tyrosine kinase | 0.01 |
| A_24_P409681 | A_24_P409681 | A_24_P409681 | Unknown | 0.01 |
| NM_003639 | A_23_P159920 | IKBKG | Inhibitor of kappa B-cells. kinase gamma | 0.01 |
| NM_001664 | A_23_P69491 | RHOA | Ras homolog gene family | 0.01 |
| NM_032378 | A_23_P31840 | EEF1D | Eukaryotic translation elongation factor 1 delta | 0.01 |
| NM_139266 | A_24_P274270 | STAT1 | Signal transducer and activator of transcription 1 | 0.01 |
| NM_078471 | A_23_P78122 | MYO18A | Myosin XVIIIA | 0.01 |
| NM_030571 | A_23_P81241 | NDFIP1 | Nedd4 family interacting protein 1 | 0.01 |
| NM_080875 | A_24_P259328 | MIB2 | Mindbomb homolog 2 | 0.01 |
| NM_138330 | A_23_P339687 | ZNF675 | Zinc finger protein 675 | 0.01 |
| NM_002342 | A_23_P53557 | LTBR | Lymphotoxin beta receptor | 0.01 |
| NM_001035005 | A_24_P271049 | LOC497661 | Putative NFkB activating protein | 0.01 |
| NM_006406 | A_23_P114232 | PRDX4 | Peroxiredoxin 4 | 0.01 |
| NM_000801 | A_32_P50522 | FKBP1A | FK506 binding protein 1A | 0.02 |
| NM_018098 | A_23_P44684 | ECT2 | Epithelial cell transforming sequence 2 oncogene | 0.02 |
| NM_178040 | A_23_P258377 | RAB6IP2 | RAB6 interacting protein 2 | 0.02 |
| NM_004425 | A_23_P160559 | ECM1 | Extracellular matrix protein 1 | 0.02 |
| NM_013254 | A_23_P44768 | TBK1 | TANK-binding kinase 1 | 0.02 |
| NM_016479 | A_23_P212475 | SCOTIN | Scotin | 0.02 |
| NM_009587 | A_32_P452655 | LGALS9 | Lectin galactoside-binding. soluble. 9 | 0.02 |
| NM_007051 | A_23_P96853 | FAF1 | Fas (TNFRSF6) associated factor 1 | 0.02 |
| NM_003270 | A_23_P171143 | TSPAN6 | Tetraspanin 6 | 0.02 |
| NM_178148 | A_23_P19257 | SLC35B2 | Solute carrier family 35 | 0.02 |
| NM_199203 | A_24_P5935 | Kua-UEV | Ubiquitin-conjugating enzyme E2 variant 1 | 0.02 |
| NM_020345 | A_23_P338519 | NKIRAS1 | NFKB inhibitor interacting Ras-like 1 | 0.02 |
| NM_003263 | A_23_P10873 | TLR1 | Toll-like receptor 1 | 0.02 |
| NM_006785 | A_23_P96008 | MALT1 | Mucosa associated lymphoid lymphoma Translocation | 0.03 |
| NM_001242 | A_23_P48088 | TNFRSF7 | TNF receptor superfamily 7 | 0.03 |
| ENST00000369797 | A_23_P161257 |  | OTTHUMP00000059187 | 0.03 |
| NM_003302 | A_24_P4054 | TRIP6 | Thyroid hormone receptor interactor 6 | 0.03 |
| NM_006068 | A_24_P17677 | TLR6 | Toll-like receptor 6 | 0.03 |
| NM_020428 | A_23_P208340 | SLC44A2 | Solute carrier family 44 | 0.03 |
| NM_001992 | A_23_P213562 | F2R | Coagulation factor II | 0.03 |
| NM_015336 | A_24_P76995 | ZDHHC17 | Zinc finger DHHC-type containing 17 | 0.04 |
| NM_016395 | A_23_P99920 | PTPLAD1 | Protein tyrosine phosphatase-like A domain containing 1 | 0.04 |
| NM_033292 | A_23_P202978 | CASP1 | Caspase 1 apoptosis-related cysteine peptidase | 0.04 |
| NM_052813 | A_23_P500433 | CARD9 | Caspase recruitment domain family | 0.04 |
| NM_021003 | A_23_P151679 | PPM1A | Protein phosphatase 1A | 0.04 |
| NM_000867 | A_23_P16953 | HTR2B | 5-hydroxytryptamine | 0.04 |
| NM_003810 | A_23_P121253 | TNFSF10 | Tumor necrosis factor (ligand) superfamily | 0.05 |
| NM_017721 | A_23_P55873 | CC2D1A | Coiled-coil and C2 domain containing 1A | 0.05 |
| NM_213590 | A_23_P87973 | RFP2 | Ret finger protein 2 | 0.06 |
| NM_006092 | A_24_P129277 | CARD4 | Caspase recruitment domain family | 0.06 |
| NM_001039661 | A_23_P397856 | TIRAP | Toll-interleukin 1 receptor | 0.07 |
| NM_148910 | A_23_P202905 | TIRAP | Toll-interleukin 1 receptor | 0.08 |
| NM_032376 | A_23_P15516 | TMEM101 | Transmembrane protein 101 | 0.08 |
|  |  |  |  |  |
| **Immune Response** | **(GO:0006955)** |  |  |  |
| **Systematic Name** | **Probe Name** | **Gene Symbol** | **Description** | **Fc** |
| NM_000584 | A_32_P87013 | IL8 | Interleukin 8 | -2.61 |
| NM_000758 | A_23_P133408 | CSF2 | Colony stimulating factor 2 | -2.27 |
| NM_001565 | A_24_P303091 | CXCL10 | Chemokine (C-X-C motif) ligand 10 | -2.26 |
| NM_004591 | A_23_P17065 | CCL20 | Chemokine (C-C motif) ligand 20 | -2.22 |
| NM_000576 | A_23_P79518 | IL1B | Interleukin 1beta | -2.13 |
| NM_000575 | A_23_P72096 | IL1A | Interleukin 1alpha | -1.62 |
| NM_002993 | A_23_P155755 | CXCL6 | Chemokine (C-X-C motif) ligand 6 | -1.59 |
| NM_005623 | A_23_P207456 | CCL8 | Chemokine (C-C motif) ligand 8 | -1.57 |
| NM_014143 | A_23_P338479 | CD274 | CD274 molecule | -1.51 |
| NM_000759 | A_23_P501754 | CSF3 | Colony stimulating factor 3 | -1.41 |
| NM_002985 | A_23_P152838 | CCL5 | Chemokine (C-C motif) ligand 5 | -1.34 |
| NM_002089 | A_23_P315364 | CXCL2 | Chemokine (C-X-C motif) ligand 2 | -1.32 |
| NM_006235 | A_23_P312920 | POU2AF1 | POU domain class2 associating factor1 | -1.32 |
| NM_001993 | A_23_P126782 | F3 | Coagulation factor III | -1.32 |
| NM_001432 | A_23_P41344 | EREG | Epiregulin | -1.32 |
| ENST00000381961 | A_23_P33643 | Q9G7C4 | Cytochrome oxidase c | -1.28 |
| NM_006273 | A_23_P78037 | CCL7 | Chemokine (C-C motif) ligand 7 | -1.26 |
| NM_030956 | A_23_P33420 | TLR10 | Toll-like receptor 10 | -1.17 |
| NM_003264 | A_23_P92499 | TLR2 | Toll-like receptor 2 | -1.12 |
| NM_005409 | A_24_P20607 | CXCL11 | Chemokine (C-X-C motif) ligand 11 | -1.11 |
| NM_001561 | A_23_P51936 | TNFRSF9 | TNF super family ligand 9 | -1.10 |
| NM_003807 | A_24_P237036 | TNFSF14 | TNF superfamily ligand 14 | -1.06 |
| NM_001252 | A_23_P119202 | TNFSF7 | TNF superfamily ligand 7 | -1.00 |
| NM_001511 | A_23_P7144 | CXCL1 | Chemokine (C-X-C motif) ligand 1 | -1.00 |
| NM_002090 | A_24_P183150 | CXCL3 | Chemokine (C-X-C motif) ligand 3 | -0.97 |
| NM_000594 | A_23_P376488 | TNF | Tumor necrosis factor | -0.93 |
| NM_002176 | A_23_P71774 | IFNB1 | Interferon. beta 1. fibroblast | -0.90 |
| NM_002996 | A_24_P381901 | CX3CL1 | Chemokine (C-X3-C motif) ligand 1 | -0.87 |
| NM_002309 | A_24_P122137 | LIF | Leukemia inhibitory factor | -0.82 |
| NM_002187 | A_23_P7560 | IL12B | Interleukin 12B | -0.80 |
| NM_000600 | A_23_P71037 | IL6 | Interleukin 6 | -0.79 |
| NM_002983 | A_23_P373017 | CCL3 | Chemokine (C-C motif) ligand 3 | -0.76 |
| NM_004233 | A_23_P70670 | CD83 | CD83 molecule | -0.76 |
| NM_004951 | A_23_P25566 | EBI2 | Epstein-Barr virus induced gene 2 | -0.71 |
| NM_052941 | A_23_P103496 | GBP4 | Guanylate binding protein 4 | -0.67 |
| NM_004833 | A_32_P44394 | AIM2 | Absent in melanoma 2 | -0.67 |
| NM_002164 | A_23_P112026 | INDO | Indoleamine-pyrrole 2,3 dioxygenase | -0.64 |
| NM_000882 | A_23_P91943 | IL12A | Interleukin 12A | -0.64 |
| NM_002982 | A_23_P89431 | CCL2 | Chemokine (C-C motif) ligand 2 | -0.60 |
| NM_006186 | A_23_P131208 | NR4A2 | Nuclear receptor subfamily 4 | -0.58 |
| NM_016584 | A_23_P76078 | IL23A | Interleukin 23, alpha subunit p19 | -0.58 |
| NM_002198 | A_23_P41765 | IRF1 | Interferon regulatory factor 1 | -0.56 |
| NM_003811 | A_23_P67224 | TNFSF9 | TNF superfamily ligand 9 | -0.56 |
| NM_181337 | A_24_P143301 | KAAG1 | Kidney associated antigen 1 | -0.56 |
| NM_002704 | A_23_P121596 | PPBP | Pro-platelet basic protein | -0.55 |
| NM_006144 | A_23_P133445 | GZMA | Granzyme A | -0.55 |
| NM_000139 | A_23_P1904 | MS4A2 | Membrane-spanning 4-domains | -0.53 |
| NM_001001437 | A_23_P321920 | CCL3L3 | Chemokine (C-C motif) ligand 3-like 3 | -0.53 |
| NM_019618 | A_23_P17053 | IL1F9 | Interleukin 1 family member 9 | -0.53 |
| NM_172140 | A_23_P337800 | IL29 | Interleukin 29 | -0.53 |
| NM_002341 | A_23_P93348 | LTB | Lymphotoxin beta | -0.53 |
| NM_002185 | A_23_P404494 | IL7R | Interleukin 7 receptor | -0.51 |
| NM_013269 | A_24_P241183 | CLEC2D | C-type lectin domain family 2 | -0.49 |
| NM_002994 | A_23_P110204 | CXCL5 | Chemokine (C-X-C motif) ligand 5 | -0.48 |
| AK022802 | A_24_P942933 | APOBEC3G | cDNA FLJ12740 fis | -0.45 |
| NM_006399 | A_23_P128974 | BATF | Basic leucine zipper transcription factor | -0.45 |
| NM_004843 | A_23_P27606 | IL27RA | Interleukin 27 receptor alpha | -0.45 |
| NM_005238 | A_23_P127525 | ETS1 | V-ets erythroblastosisvirus E26 oncogene homolog 1 | -0.44 |
| NM_025239 | A_23_P94412 | PDCD1LG2 | Programmed cell death 1 ligand 2 | -0.44 |
| NM_003855 | A_24_P208567 | IL18R1 | Interleukin 18 receptor 1 | -0.41 |
| NM_004244 | A_23_P33723 | CD163 | CD163 molecule | -0.41 |
| NM_004258 | A_24_P131066 | IGSF2 | Immunoglobulin superfamily | -0.41 |
| NM_002053 | A_32_P107372 | GBP1 | Guanylate binding protein 1 | -0.41 |
| NM_000397 | A_23_P217258 | CYBB | Cytochrome b-245 beta polypeptide | -0.38 |
| NM_002986 | A_23_P66635 | CCL11 | Chemokine (C-C motif) ligand 11 | -0.38 |
| NM_004810 | A_24_P10884 | GRAP2 | GRB2-related adaptor protein 2 | -0.37 |
| NM_000064 | A_23_P101407 | C3 | Complement component 3 | -0.37 |
| NM_005191 | A_23_P155632 | CD80 | CD80 molecule | -0.36 |
| NM_005408 | A_24_P125335 | CCL13 | Chemokine (C-C motif) ligand 13 | -0.35 |
| NM_003998 | A_23_P30024 | NFKB1 | Nuclear factor of kappa B-cells 1 | -0.35 |
|  |  |  |  |  |
| **Inflammatory response** | **(GO:0006954)** |  |  |  |
| **Systematic Name** | **Probe Name** | **Gene Symbol** | **Description** | **Fc** |
| NM_000584 | A_32_P87013 | IL8 | Interleukin 8 | -2.61 |
| NM_001565 | A_24_P303091 | CXCL10 | Chemokine (C-X-C motif) ligand 10 | -2.26 |
| NM_004591 | A_23_P17065 | CCL20 | Chemokine (C-C motif) ligand 20 | -2.22 |
| NM_000576 | A_23_P79518 | IL1B | Interleukin 1beta | -2.13 |
| NM_000450 | A_23_P97112 | SELE | Selectin E | -1.67 |
| NM_000575 | A_23_P72096 | IL1A | Interleukin 1. alpha | -1.62 |
| NM_002993 | A_23_P155755 | CXCL6 | Chemokine (C-X-C motif) ligand 6 | -1.59 |
| NM_005623 | A_23_P207456 | CCL8 | Chemokine (C-C motif) ligand 8 | -1.57 |
| NM_002985 | A_23_P152838 | CCL5 | Chemokine (C-C motif) ligand 5 | -1.34 |
| NM_002089 | A_23_P315364 | CXCL2 | Chemokine (C-X-C motif) ligand 2 | -1.32 |
| NM_006273 | A_23_P78037 | CCL7 | Chemokine (C-C motif) ligand 7 | -1.26 |
| NM_030956 | A_23_P33420 | TLR10 | Toll-like receptor 10 | -1.17 |
| NM_003264 | A_23_P92499 | TLR2 | Toll-like receptor 2 | -1.12 |
| NM_005409 | A_24_P20607 | CXCL11 | Chemokine (C-X-C motif) ligand 11 | -1.11 |
| NM_014589 | A_23_P114857 | PLA2G2E | Phospholipase A2 group IIE | -1.08 |
| NM_001511 | A_23_P7144 | CXCL1 | Chemokine (C-X-C motif) ligand 1 | -1.00 |
| NM_002090 | A_24_P183150 | CXCL3 | Chemokine (C-X-C motif) ligand 3 | -0.97 |
| NM_001570 | A_23_P80635 | IRAK2 | Interleukin-1 receptor-associated kinase 2 | -0.90 |
| NM_000963 | A_24_P250922 | PTGS2 | Prostaglandin-endoperoxide synthase 2 | -0.88 |
| NM_002996 | A_24_P381901 | CX3CL1 | Chemokine (C-X3-C motif) ligand 1 | -0.87 |
| NM_003734 | A_23_P426305 | AOC3 | Amine oxidase, copper containing 3 | -0.83 |
| NM_000600 | A_23_P71037 | IL6 | Interleukin 6 | -0.79 |
| NM_000675 | A_23_P109436 | ADORA2A | Adenosine A2a receptor | -0.76 |
| NM_002983 | A_23_P373017 | CCL3 | Chemokine (C-C motif) ligand 3 | -0.76 |
| NM_003182 | A_23_P215283 | TAC1 | Tachykinin. precursor 1 | -0.70 |
| NM_001200 | A_23_P143331 | BMP2 | Bone morphogenetic protein 2 | -0.60 |
| NM_002982 | A_23_P89431 | CCL2 | Chemokine (C-C motif) ligand 2 | -0.60 |
| NM_016584 | A_23_P76078 | IL23A | Interleukin 23, alpha subunit p19 | -0.58 |
| NM_001001437 | A_23_P321920 | CCL3L3 | Chemokine (C-C motif) ligand 3-like 3 | -0.53 |
| NM_023068 | A_23_P17481 | SIGLEC1 | Sialic acid binding Ig-like lectin 1 | -0.53 |
| NM_007115 | A_23_P165624 | TNFAIP6 | Tumor necrosis factor. alpha-induced protein 6 | -0.50 |
| NM_002994 | A_23_P110204 | CXCL5 | Chemokine (C-X-C motif) ligand 5 | -0.48 |
| NM_004244 | A_23_P33723 | CD163 | CD163 molecule | -0.41 |
| NM_138938 | A_23_P119936 | REG3A | Regenerating islet-derived 3 alpha | -0.39 |
| NM_001066 | A_24_P54174 | TNFRSF1B | Tumor necrosis factor receptor superfamily | -0.39 |
| NM_058176 | A_23_P215577 | HDAC9 | Histone deacetylase 9 | -0.39 |
| NM_000397 | A_23_P217258 | CYBB | Cytochrome b-245, beta polypeptide | -0.38 |
| NM_014707 | A_23_P404162 | HDAC9 | Histone deacetylase 9 | -0.38 |
| NM_000710 | A_23_P128744 | BDKRB1 | Bradykinin receptor B1 | -0.38 |
| NM_002986 | A_23_P66635 | CCL11 | Chemokine (C-C motif) ligand 11 | -0.38 |
| NM_000064 | A_23_P101407 | C3 | Complement component 3 | -0.37 |
| NM_005408 | A_24_P125335 | CCL13 | Chemokine (C-C motif) ligand 13 | -0.35 |
| NM_003998 | A_23_P30024 | NFKB1 | Nuclear factor of kappa B-cells 1 | -0.35 |
| NM_012275 | A_23_P39856 | IL1F5 | Interleukin 1 family | -0.33 |
| NM_015991 | A_24_P222655 | C1QA | Complement component 1q subcomponent A chain | -0.32 |
| NM_003152 | A_23_P207367 | STAT5A | Signal transducer and activator of transcription 5A | -0.32 |
| NM_145912 | A_23_P17911 | NFAM1 | NFAT activating protein with ITAM motif 1 | -0.31 |
| NM_002984 | A_23_P207564 | CCL4 | Chemokine (C-C motif) ligand 4 | -0.30 |
| NM_003821 | A_23_P252106 | RIPK2 | Receptor-interacting serine-threonine kinase 2 | -0.30 |
| NM_004895 | A_23_P9883 | CIAS1 | Cold autoinflammatory syndrome 1 | -0.28 |
| NM_002981 | A_23_P49759 | CCL1 | Chemokine (C-C motif) ligand 1 | -0.27 |
| NM_145637 | A_23_P211488 | APOL2 | Apolipoprotein L. 2 | -0.27 |
| NM_001203 | A_24_P63380 | BMPR1B | Bone morphogenetic protein receptor. type IB | -0.27 |
| NM_022718 | A_23_P376557 | MMP25 | Matrix metallopeptidase 25 | -0.26 |
| NM_000572 | A_23_P126735 | IL10 | Interleukin 10 | -0.25 |
| NM_000066 | A_23_P35252 | C8B | Complement component 8. beta polypeptide | -0.25 |
| NM_173842 | A_23_P209995 | IL1RN | Interleukin 1 receptor antagonist | -0.25 |
| NM_000647 | A_23_P212354 | CCR2 | Chemokine (C-C motif) receptor 2 | -0.25 |
| NM_002416 | A_23_P18452 | CXCL9 | Chemokine (C-X-C motif) ligand 9 | -0.24 |
| NM_001710 | A_23_P156687 | CFB | Complement factor B | -0.23 |
| NM_000623 | A_23_P304897 | BDKRB2 | Bradykinin receptor B2 | -0.21 |
| NM_001140 | A_23_P55373 | ALOX15 | Arachidonate 15-lipoxygenase | -0.21 |
|  |  |  |  |  |
| **Leukocyte Chemotaxis** | **(GO:0030595)** |  |  |  |
| **Systematic Name** | **Probe Name** | **Gene Symbol** | **Description** | **Fc** |
| NM_000584 | A_32_P87013 | IL8 | Interleukin 8 | -2.61 |
| NM_000576 | A_23_P79518 | IL1B | Interleukin-1beta | -2.13 |
|  |  |  |  |  |
| **Locomotory Behavior** | **(GO:0007626)** |  |  |  |
| **Systematic Name** | **Probe Name** | **Gene Symbol** | **Description** | **Fc** |
| NM_000584 | A_32_P87013 | IL8 | Interleukin 8 | -2.61 |
| NM_001565 | A_24_P303091 | CXCL10 | Chemokine (C-X-C motif) ligand 10 | -2.26 |
| NM_004591 | A_23_P17065 | CCL20 | Chemokine (C-C motif) ligand 20 | -2.22 |
| NM_000576 | A_23_P79518 | IL1B | Interleukin 1beta | -2.13 |
| NM_000575 | A_23_P72096 | IL1A | Interleukin 1alpha | -1.62 |
| NM_002993 | A_23_P155755 | CXCL6 | Chemokine (C-X-C motif) ligand 6 | -1.59 |
| NM_005623 | A_23_P207456 | CCL8 | Chemokine (C-C motif) ligand 8 | -1.57 |
| NM_002985 | A_23_P152838 | CCL5 | Chemokine (C-C motif) ligand 5 | -1.34 |
| NM_002089 | A_23_P315364 | CXCL2 | Chemokine (C-X-C motif) ligand 2 | -1.32 |
| NM_006273 | A_23_P78037 | CCL7 | Chemokine (C-C motif) ligand 7 | -1.26 |
| NM_005409 | A_24_P20607 | CXCL11 | Chemokine (C-X-C motif) ligand 11 | -1.11 |
| NM_001511 | A_23_P7144 | CXCL1 | Chemokine (C-X-C motif) ligand 1 | -1.00 |
| NM_002090 | A_24_P183150 | CXCL3 | Chemokine (C-X-C motif) ligand 3 | -0.97 |
|  |  |  |  |  |
| **Regulation of Apoptosis**  **(GO:0043066)** | |  |  |  |
| **Systematic Name** | **Probe Name** | **Gene Symbol** | **Description** | **Fc** |
| NM_000758 | A_23_P133408 | CSF2 | **Colony stimulating factor 2** | -2.27 |
| NM_000575 | A_23_P72096 | IL1A | Interleukin 1alpha | -1.62 |
| NM_000594 | A_23_P376488 | TNF | Tumor necrosis factor | -0.93 |
| NM_006290 | A_24_P157926 | TNFAIP3 | Tumor necrosis factor | -0.85 |
| NM_002575 | A_24_P245379 | SERPINB2 | Serpin peptidase inhibitor-clade B | -0.83 |
| NM_000600 | A_23_P71037 | IL6 | Interleukin 6 | -0.79 |
| NM_153694 | A_23_P76374 | SYCP3 | Synaptonemal complex protein 3 | -0.69 |
| NM_001165 | A_23_P98350 | BIRC3 | Baculoviral IAP repeat-containing 3 | -0.61 |
| NM_002982 | A_23_P89431 | CCL2 | Chemokine (C-C motif) ligand 2 | -0.60 |
| NM_004923 | A_23_P161507 | MTL5 | Metallothionein-like 5 testis-specific | -0.57 |
| NM_004049 | A_23_P152002 | BCL2A1 | BCL2-related protein A1 | -0.54 |
| NM_003897 | A_23_P42257 | IER3 | Immediate early response 3 | -0.52 |
| ENST00000299502 | A_23_P153185 |  | cDNAclone CS0DH002YJ05, T cells | -0.50 |
| NM_003955 | A_23_P207058 | SOCS3 | Suppressor of cytokine signaling 3 | -0.41 |
| NM_003998 | A_23_P30024 | NFKB1 | Nuclear factor of kappa B-cells 1 | -0.35 |
| NM_013246 | A_23_P138760 | CLCF1 | Cardiotrophin-like cytokine factor 1 | -0.33 |
| NM_000312 | A_23_P40096 | PROC | Protein C | -0.32 |
| NM_003152 | A_23_P207367 | STAT5A | Signal transducer and activator of transcription 5A | -0.32 |
| NM_014350 | A_32_P219520 | TNFAIP8 | Tumor necrosis factor alpha-induced protein 8 | -0.32 |
| AF009616 | A_23_P209394 | CFLAR | FLAME-1 | -0.29 |
| NM_000880 | A_23_P8961 | IL7 | Interleukin 7 | -0.26 |
| NM_000572 | A_23_P126735 | IL10 | Interleukin 10 | -0.25 |
| NM_005178 | A_23_P4662 | BCL3 | B-cell CLL/lymphoma 3 | -0.25 |
| NM_000477 | A_23_P257834 | ALB | Albumin | -0.25 |
| NM_003879 | A_24_P120115 | CFLAR | CASP8 and FADD-like apoptosis regulator | -0.23 |
| A_23_P170719 | A_23_P170719 | A_23_P170719 | Unknown | -0.19 |
| NM_016569 | A_23_P13772 | TBX3 | T-box 3 | -0.18 |
| NM_139314 | A_23_P159325 | ANGPTL4 | Angiopoietin-like 4 | -0.18 |
| AJ008005 | A_23_P106174 | PSEN1 | PSN1 gene alternative transcript | -0.18 |
| NM_021972 | A_23_P38106 | SPHK1 | Sphingosine kinase 1 | -0.17 |
| NM_014336 | A_23_P100935 | AIPL1 | Aryl hydrocarbon receptor interacting protein-like 1 | -0.16 |
| NM_003840 | A_23_P95417 | TNFRSF10D | Tumor necrosis factor receptor superfamily | -0.16 |
| NM_005345 | A_23_P111132 | HSPA1A | Heat shock 70kDa protein 1A | -0.16 |
| NM_017617 | A_23_P60387 | NOTCH1 | Notch homolog 1 translocation-associated | -0.16 |
| NM_005734 | A_23_P422809 | HIPK3 | Homeodomain interacting protein kinase 3 | -0.15 |
| NM_005194 | A_23_P411296 | CEBPB | CCAAT/enhancer binding protein | -0.15 |
| NM_170735 | A_23_P127891 | BDNF | Brain-derived neurotrophic factor | -0.15 |
| NM_000551 | A_24_P134942 | VHL | Von Hippel-Lindau tumor suppressor | -0.13 |
| NM_138931 | A_23_P57856 | BCL6 | B-cell CLL/lymphoma 6 | -0.13 |
| XM_942822 | A_32_P703 | LOC646626 | Hypothetical protein LOC647393 | -0.13 |
| NM_000043 | A_23_P63896 | FAS | Fas | -0.13 |
| NM_001166 | A_24_P115774 | BIRC2 | Baculoviral IAP repeat-containing 2 | -0.12 |
| NM_012068 | A_23_P119337 | ATF5 | Activating transcription factor 5 | -0.12 |
| ENST00000374189 | A_23_P202540 |  | Unknown | -0.11 |
| BC020868 | A_24_P342178 | STAT5B | Signal transducer and activator of transcription 5B | -0.11 |
| NM_021960 | A_24_P319635 | MCL1 | Myeloid cell leukemia sequence 1 | -0.10 |
| NM_007308 | A_23_P29939 | SNCA | Synuclein alpha | -0.10 |
| NM_003877 | A_23_P128215 | SOCS2 | Suppressor of cytokine signaling 2 | -0.10 |
| NM_001025366 | A_23_P81805 | VEGF | Vascular endothelial growth factor | -0.09 |
| NM_003921 | A_23_P115286 | BCL10 | B-cell CLL/lymphoma 10 | -0.09 |
| NM_012448 | A_23_P100788 | STAT5B | Signal transducer and activator of transcription 5B | -0.08 |
| NM_000546 | A_23_P26810 | TP53 | Tumor protein p53 | -0.08 |
| NM_023111 | A_23_P219105 | FGFR1 | Fibroblast growth factor receptor 1 | -0.08 |
| NM_000875 | A_23_P205986 | IGF1R | Insulin-like growth factor 1 receptor | -0.07 |
|  |  |  |  |  |
| **Regulation of cell proliferation** | **(GO:0042127)** |  |  |  |
| **Systematic Name** | **Probe Name** | **Gene Symbol** | **Description** | **Fc** |
| NM_000584 | A_32_P87013 | IL8 | Interleukin 8 | -2.61 |
| NM_000758 | A_23_P133408 | CSF2 | Colony stimulating factor 2 | -2.27 |
| NM_001565 | A_24_P303091 | CXCL10 | Chemokine (C-X-C motif) ligand 10 | -2.26 |
| NM_000576 | A_23_P79518 | IL1B | Interleukin-1beta | -2.13 |
| NM_001965 | A_23_P380318 | EGR4 | Early growth response 4 | -2.07 |
| NM_000575 | A_23_P72096 | IL1A | Interleukin-1alpha | -1.62 |
| NM_000759 | A_23_P501754 | CSF3 | Colony stimulating factor 3 | -1.41 |
| NM_001432 | A_23_P41344 | EREG | Epiregulin | -1.32 |
| NM_001561 | A_23_P51936 | TNFRSF9 | TNF superfamily ligand 9 | -1.10 |
| NM_001511 | A_23_P7144 | CXCL1 | Chemokine (C-X-C motif) ligand 1 | -1.00 |
| AF118092 | A_24_P359491 | AF118092 | PRO2061 | -0.96 |
| NM_000594 | A_23_P376488 | TNF | Tumor necrosis factor | -0.93 |
| NM_002176 | A_23_P71774 | IFNB1 | Interferon-beta 1 | -0.90 |
| NM_001945 | A_23_P213944 | HBEGF | Heparin-binding EGF-like Growth F | -0.83 |
| NM_002309 | A_24_P122137 | LIF | Leukemia inhibitory factor | -0.82 |
| NM_002187 | A_23_P7560 | IL12B | Interleukin 12B | -0.80 |
| NM_000600 | A_23_P71037 | IL6 | Interleukin 6 | -0.79 |
|  |  |  |  |  |
|  |  |  |  |  |
| **Response to Wounding** | **(GO:0009611)** |  |  |  |
| **Systematic Name** | **Probe Name** | **Gene Symbol** | **Description** | **Fc** |
| NM_000584 | A_32_P87013 | IL8 | Interleukin 8 | - 2.61 |
| NM_001565 | A_24_P303091 | CXCL10 | Chemokine (C-X-C motif) ligand 10 | -2.26 |
| NM_004591 | A_23_P17065 | CCL20 | Chemokine (C-C motif) ligand 20 | -2.22 |
| NM_000576 | A_23_P79518 | IL1B | Interleukin-1beta | -2.13 |
| NM_000450 | A_23_P97112 | SELE | Selectin E | -1.67 |
| NM_000575 | A_23_P72096 | IL1A | Interleukin-1alpha | -1.62 |
| NM_002993 | A_23_P155755 | CXCL6 | Chemokine (C-X-C motif) ligand 6 | -1.59 |
| NM_005623 | A_23_P207456 | CCL8 | Chemokine (C-C motif) ligand 8 | -1.57 |
| NM_002985 | A_23_P152838 | CCL5 | Chemokine (C-C motif) ligand 5 | -1.34 |
| NM_002089 | A_23_P315364 | CXCL2 | Chemokine (C-X-C motif) ligand 2 | -1.32 |
| NM_001993 | A_23_P126782 | F3 | Coagulation factor III | -1.32 |
| NM_001432 | A_23_P41344 | EREG | Epiregulin | -1.32 |
| NM_006273 | A_23_P78037 | CCL7 | Chemokine (C-C motif) ligand 7 | -1.26 |
| NM_030956 | A_23_P33420 | TLR10 | Toll-like receptor 10 | -1.17 |
| NM_003264 | A_23_P92499 | TLR2 | Toll-like receptor 2 | -1.12 |
| NM_005409 | A_24_P20607 | CXCL11 | Chemokine (C-X-C motif) ligand 11 | -1.11 |
| NM_014589 | A_23_P114857 | PLA2G2E | Phospholipase A2. group IIE | -1.08 |
| NM_001511 | A_23_P7144 | CXCL1 | Chemokine (C-X-C motif) ligand 1 | -1.00 |
| NM_002090 | A_24_P183150 | CXCL3 | Chemokine (C-X-C motif) ligand 3 | -0.97 |
| AF118092 | A_24_P359491 | AF118092 | PRO2061 | -0.96 |
| NM_001570 | A_23_P80635 | IRAK2 | Interleukin-1 receptor-associated kinase 2 | -0.90 |
| NM_000963 | A_24_P250922 | PTGS2 | Prostaglandin-endoperoxide synthase 2 | -0.88 |
| NM_002996 | A_24_P381901 | CX3CL1 | Chemokine (C-X3-C motif) ligand 1 | -0.87 |
| ENST00000248076 | A_24_P228470 | PAR4 | Protease-activated receptor 4 | -0.83 |
| NM_001945 | A_23_P213944 | HBEGF | Heparin-binding EGF-like Growth F | -0.83 |
| NM_003734 | A_23_P426305 | AOC3 | Amine oxidase copper containing 3 | -0.83 |
| NM_000600 | A_23_P71037 | IL6 | Interleukin 6 | -0.79 |
| ENST00000222543 | A_24_P95070 |  | cDNA FLJ26323 fis. clone HRT00813 | -0.76 |
| NM_000675 | A_23_P109436 | ADORA2A | Adenosine A2a receptor | -0.76 |
| NM_002983 | A_23_P373017 | CCL3 | Chemokine (C-C motif) ligand 3 | -0.76 |
| NM_003182 | A_23_P215283 | TAC1 | Tachykinin. precursor 1 | -0.70 |
| J02940 | A_23_P152926 | GP1BA | Human platelet glycoprotein Ib alpha chain | -0.69 |
| NM_001200 | A_23_P143331 | BMP2 | Bone morphogenetic protein 2 | -0.60 |
| NM_002982 | A_23_P89431 | CCL2 | Chemokine (C-C motif) ligand 2 | -0.60 |
| NM_016584 | A_23_P76078 | IL23A | Interleukin 23. alpha subunit p19 | -0.58 |
| NM_005242 | A_23_P58835 | F2RL1 | Coagulation factor II | -0.55 |
| NM_001001437 | A_23_P321920 | CCL3L3 | Chemokine (C-C motif) ligand 3-like 3 | -0.53 |
| NM_023068 | A_23_P17481 | SIGLEC1 | Sialic acid binding Ig-like lectin 1 | -0.53 |
| NM_004200 | A_23_P161935 | SYT7 | Synaptotagmin VII | -0.51 |
| NM_007115 | A_23_P165624 | TNFAIP6 | Tumor necrosis factor. alpha-induced protein 6 | -0.50 |
| NM_002994 | A_23_P110204 | CXCL5 | Chemokine (C-X-C motif) ligand 5 | -0.48 |
| NM_002608 | A_24_P339944 | PDGFB | Platelet-derived growth factor beta polypeptide | -0.46 |
| NM_004244 | A_23_P33723 | CD163 | CD163 molecule | -0.41 |
| NM_138938 | A_23_P119936 | REG3A | Regenerating islet-derived 3 alpha | -0.39 |
| NM_001066 | A_24_P54174 | TNFRSF1B | Tumor necrosis factor receptor superfamily | -0.39 |
| NM_058176 | A_23_P215577 | HDAC9 | Histone deacetylase 9 | -0.39 |
| NM_000397 | A_23_P217258 | CYBB | Cytochrome b-245. beta polypeptide | -0.38 |
| NM_014707 | A_23_P404162 | HDAC9 | Histone deacetylase 9 | -0.38 |
| NM_000710 | A_23_P128744 | BDKRB1 | Bradykinin receptor B1 | -0.38 |
| NM_002986 | A_23_P66635 | CCL11 | Chemokine (C-C motif) ligand 11 | -0.38 |
| NM_000064 | A_23_P101407 | C3 | Complement component 3 | -0.37 |
| NM_005408 | A_24_P125335 | CCL13 | Chemokine (C-C motif) ligand 13 | -0.35 |
| NM_003998 | A_23_P30024 | NFKB1 | Nuclear factor of kappa B-cells 1 | -0.35 |
| NM_000641 | A_23_P67169 | IL11 | Interleukin 11 | -0.34 |
| NM_012275 | A_23_P39856 | IL1F5 | Interleukin 1 family member 5 | -0.33 |
| NM_015991 | A_24_P222655 | C1QA | Complement component 1q subcomponent | -0.32 |
| NM_000312 | A_23_P40096 | PROC | Protein C | -0.32 |
| NM_003152 | A_23_P207367 | STAT5A | Signal transducer and activator of transcription 5A | -0.32 |
| NM_145912 | A_23_P17911 | NFAM1 | NFAT activating protein with ITAM motif 1 | -0.31 |
| NM_002984 | A_23_P207564 | CCL4 | Chemokine (C-C motif) ligand 4 | -0.30 |
| NM_003821 | A_23_P252106 | RIPK2 | Receptor-interacting serine-threonine kinase 2 | -0.30 |
| NM_000602 | A_24_P158089 | SERPINE1 | Serpin peptidase inhibitor clade E | -0.30 |
| NM_004895 | A_23_P9883 | CIAS1 | Cold autoinflammatory syndrome 1 | -0.28 |
| NM_002981 | A_23_P49759 | CCL1 | Chemokine (C-C motif) ligand 1 | -0.27 |
| NM_145637 | A_23_P211488 | APOL2 | Apolipoprotein L2 | -0.27 |
| NM_000488 | A_23_P114626 | SERPINC1 | Serpin peptidase inhibitor clade C member 1 | -0.27 |
| NM_000361 | A_23_P91390 | THBD | Thrombomodulin | -0.27 |
| NM_001203 | A_24_P63380 | BMPR1B | Bone morphogenetic protein receptor type IB | -0.27 |
| NM_004148 | A_23_P169137 | NINJ1 | Ninjurin 1 | -0.26 |
| NM_022718 | A_23_P376557 | MMP25 | Matrix metallopeptidase 25 | -0.26 |
| NM_000572 | A_23_P126735 | IL10 | Interleukin 10 | -0.25 |
| NM_000066 | A_23_P35252 | C8B | Complement component 8beta polypeptide | -0.25 |
| NM_173842 | A_23_P209995 | IL1RN | Interleukin 1 receptor antagonist | -0.25 |
| NM_000647 | A_23_P212354 | CCR2 | Chemokine (C-C motif) receptor 2 | -0.25 |
| NM_002416 | A_23_P18452 | CXCL9 | Chemokine (C-X-C motif) ligand 9 | -0.24 |
| NM_001710 | A_23_P156687 | CFB | Complement factor B | -0.23 |
| NM_006528 | A_23_P393620 | TFPI2 | Tissue factor pathway inhibitor 2 | -0.22 |
| NM_000623 | A_23_P304897 | BDKRB2 | Bradykinin receptor B2 | -0.21 |
| NM_000212 | A_23_P38519 | ITGB3 | Integrin-beta 3 | -0.21 |
| NM_001140 | A_23_P55373 | ALOX15 | Arachidonate 15-lipoxygenase | -0.21 |
